# Targeting anemia-induced CD71^+^ reticulocytes protects mice from *Plasmodium* infection

**DOI:** 10.1128/iai.00093-25

**Published:** 2025-07-01

**Authors:** Sareh Zeydabadinejad, Jong Sung Anthony Kim, Anna Zheng, Mrunmayee Rajendra Kandalgaonkar, Prince Boakye Ababio, Amira Gohara, Matam Vijay-Kumar, Beng San Yeoh, Piu Saha

**Affiliations:** 1 Center for Hypertension and Precision Medicine, Department of Physiology and Pharmacology, University of Toledo College of Medicine and Life Sciences 89021, Toledo, Ohio, USA; 2 The University of Toledo College of Medicine and Life Sciences 89021, Toledo, Ohio, USA; 3 Department of Pathology, University of Toledo Medical Center 70257, Toledo, Ohio, USA; University of California Davis, Davis, California, USA

**Keywords:** malaria, RBC, TER-cells, phenylhydrazine, phlebotomy

## Abstract

Malaria, caused by *Plasmodium* spp., is a global health concern linked to anemia and increased mortality. Compensatory erythropoiesis seen during acute anemia results in an increased circulating reticulocyte count (i.e., immature RBC), a key factor in understanding the relationship between pre-existing anemia and *Plasmodium* burden. Reticulocytes in mice are marked by transferrin receptor (CD71^+^) and glycophorin A-associated protein (Ter119^+^). To model acute anemia with increased reticulocytes, C57BL/6J mice were either bled (i.e., phlebotomized) or administered phenylhydrazine before being infected with *Plasmodium yoelii* (*P. yoelii*), a mouse-specific strain with a preference for reticulocytes. In *P. yoelii*-infected anemic mice, we observed heightened parasitemia and significant body weight loss compared with non-anemic *P. yoelii*-infected mice. Additionally, serum inflammatory cytokines, erythropoietin, and liver injury markers, along with hemozoin deposition, significantly increased in anemic *P. yoelii*-infected mice. Blood transfusion from healthy normal donors to *P. yoelii*-infected anemic recipient mice ameliorated anemia by reducing overall reticulocyte count and increasing mature RBC count. Blood transfusion rescued body weight loss, decreased parasitemia, and reduced serum erythropoietin levels. Finally, to confirm the role of reticulocytes in *P. yoelii* infection, reticulocytes were depleted using anti-CD71 monoclonal antibody in *P. yoelii*-infected mice. We observed improvement in hematologic parameters and stark reduction in parasitemia in both pre-existing anemic and non-anemic *P. yoelii*-infected mice. Collectively, our results suggest that pre-existing anemia may increase the risk of *Plasmodium* infection due to the greater reticulocyte population. Anti-CD71 treatment in *Plasmodium* infection may offer a novel therapeutic strategy to combat *Plasmodium* infection and malaria.

## INTRODUCTION

Malaria is a vector-borne disease caused by the protozoan parasite *Plasmodium*. Despite the increasing efforts to minimize its public health burden in malaria-endemic regions, *Plasmodium* continues to infect over 245 million people each year globally and claimed 619,000 lives in 2021. *Plasmodium falciparum* and *Plasmodium vivax* are of the greatest concern, accounting for 95% of human malaria cases. *P. falciparum* is responsible for most malaria mortality, while *P. vivax* is the most frequent cause of malaria. The disparity in disease severity caused by the two strains is, in part, due to differences in their inclinations to infect immature RBCs (reticulocytes) versus mature RBCs (*aka* erythrocytes). Whereas *P. vivax* could preferably infect reticulocytes (1), *P. falciparum* infects both reticulocytes and erythrocytes (2). Despite the less restrictive host tropism of *P. falciparum*, the parasite exhibits a strong preference to infect reticulocytes when available (2). Thus, having a better understanding of the RBC tropism of *Plasmodium* spp. would help in the development of anti-malarial therapeutics tailored specifically to prevent RBC parasitization.

Reticulocytes are distinctively characterized by their surface co-expression of the glycophorin A-associated protein, Ter119 (i.e., erythroid lineage marker), and the transferrin receptor, CD71, that plays a key role in iron uptake (2–4). As the reticulocytes reach terminal maturation, they not only remove their nucleus and organelles but also shed CD71 from their cell membrane (5). Circulating reticulocytes, which retain CD71, represent only a small fraction of the total RBC pool, as most RBCs mature in the bone marrow before they are released into the bloodstream (5). However, in the event of blood loss and/or anemia, the bone marrow is unable to adequately replenish the RBC pool, and this activates the compensatory stress erythropoiesis (*alias* extramedullary hematopoiesis) in the spleen and liver. The RBCs produced in this manner are released prematurely, thus substantially elevating reticulocyte counts in circulation. Whether such an increase in circulating reticulocytes could predispose mice to severe malaria, particularly if it occurs before *Plasmodium* infection, remains poorly understood and warrants further study.

Severe anemia significantly contributes to malaria-associated pathology, particularly among children in sub-Saharan Africa, which accounts for about 95% of global malaria cases, making it a major health concern (6–8). The onset of malarial anemia can be attributed to the lysis of parasitized RBCs and the malaria-associated suppression of bone marrow erythropoiesis (i.e., ineffective erythropoiesis) (8–10). Of note, most of the studies on malarial anemia are heavily focused on anemia being a consequence of the disease, rightfully considering its role as a co-morbidity. In contrast, there are few studies that sought to determine whether pre-existing anemia could also play a causal role in increasing the severity of *Plasmodium* infection. It is reasonable to consider, for instance, that individuals with higher reticulocyte count due to a pre-existing anemia may exhibit more severe infection with *Plasmodium* spp. that prefer to infect reticulocytes. Thus, in this study, we employed mouse models to investigate whether acute anemia that arises due to phlebotomy and/or phenylhydrazine (PHZ) could predispose mice to infection by *Plasmodium yoelii* (i.e., a mouse-specific strain). Furthermore, we explored whether targeted depletion of reticulocytes via the use of anti-CD71 monoclonal antibody (α-CD71 mAb) could protect anemic mice from malaria.

Here, we showed that phlebotomy and PHZ-induced anemia increased circulating reticulocytes and heightened the severity of *P. yoelii* infection in mice. Malaria severity in pre-anemic mice was exacerbated as denoted by the increased parasitemia and markers of liver injury (serum total bile acids [TBA], alanine transaminase [ALT]), multi-organ injury (aspartate transaminase [AST]) and inflammation (lipocalin 2 [Lcn2], serum amyloid A [SAA]). Transfusion of RBCs from healthy normal mice into anemic mice infected with *P. yoelii* decreased the abundance of circulating reticulocytes and conferred protection against body weight loss, parasitemia, and other indices of malaria severity. Further depletion of CD71^+^ reticulocytes using α-CD71 mAb in *P. yoelii*-infected mice substantially improved the hematological parameters (RBC, hemoglobin, and hematocrit), immune cell counts (WBC, monocytes, lymphocytes, and neutrophils), and markedly reduced parasitemia in both pre-existing anemic and non-anemic mice. Collectively, our findings underscore the critical role of reticulocytes in malaria pathogenesis and propose novel therapeutic opportunities by targeting reticulocyte populations.

## RESULTS

### Phlebotomy-induced anemia increased Ter119^+^ CD71^+^ reticulocytes and showed greater *Plasmodium* infection

Frequent blood draws in a clinical setting can result in phlebotomy-induced anemia (i.e., nosocomial anemia) (11, 12). To model such anemia in mice, we performed submandibular bleeding on C57BL/6J male mice for two consecutive days. Complete blood count (CBC) analyses at 24 h after the second blood draw confirmed that phlebotomized mice had a significant decrease in RBC count, hemoglobin, and hematocrit compared to control mice (Fig. S1A through C). Phlebotomized mice also displayed a decrease in hemoglobin content as indicated by mean corpuscular hemoglobin (MCH) and mean corpuscular hemoglobin concentration (MCHC), but no difference in mean corpuscular volume (MCV; Fig. S1D through F) compared to control mice. Total white blood cell (WBC) count was moderately reduced, whereas monocyte and neutrophil counts were significantly reduced in phlebotomized mice (Fig. S1G through I). Lymphocyte count, however, was comparable between phlebotomized and control mice (Fig. S1J). These results indicate that the mice in the phlebotomized group were anemic with compromised innate immune cells.

**Fig 1:**
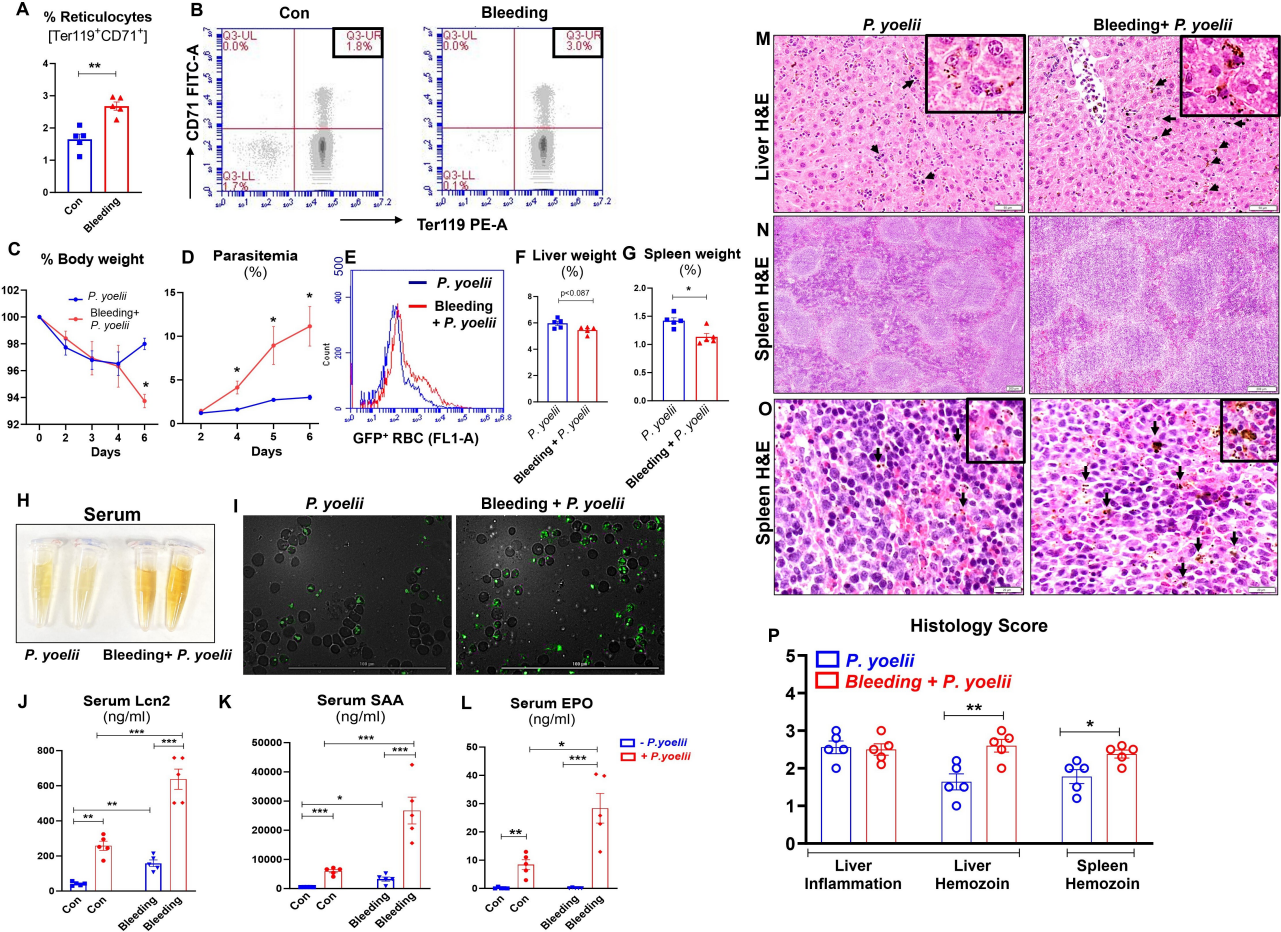
Phlebotomy-induced anemic mice had heightened reticulocytes and exhibited more severe *P. yoelii* infection. Blood (~200 µL/day) was collected from 10-week-old male WT mice (*n* = 5/group) over two consecutive days via submandibular bleeding to induce anemia. Flow cytometry analysis was performed on the blood to assess reticulocytes (Ter119^+^CD71^+^ cells) 24 h after the second blood draw and before infection with *P. yoelii*. (**A**) Bar graphs show the % reticulocytes. (**B**) Representative dot plots of reticulocytes. Mice were infected with *P. yoelii* 24 h after the second blood draw and euthanized on day 6 post-infection (p.i.), (**C**) % body weight, (**D**) % parasitemia, as indicated by green fluorescent protein (GFP)-positive RBC, was measured by flow cytometry during the infection. Quantification of parasitemia (% GFP^+^ RBC) represents GFP-*P. yoelii*-infected RBC. (**E**) Flow cytometry representation of % GFP-*P. yoelii*-infected RBC in histograms, (**F**) % liver weight, (**G**) % spleen weight, (**H**) serum color on day 6 p.i., (**I**) representative images of GFP-*P. yoelii*-infected RBC visualized under a fluorescence microscope in infected mice on day 6 p.i. Scale bar = 100 µm; magnification = 40× (bright field and FITC). Serum samples were analyzed for cytokines: (**J–L**) Lcn2, SAA, and erythropoietin (EPO) measured by enzyme-linked immunosorbent assay (ELISA). Liver and spleen sections were processed for Hematoxylin-eosin (H&E) staining to assess histopathological changes. (**M**) Liver histology, bars = 50 µm. (**N–O**) Spleen histology shown at 4× (bars = 200 µm) and 40× (bars = 20 µm) magnification. Black arrows indicate parasitized red blood cells (PRBCs) and hemozoin pigment sequestration. Inset in M and O shows hemozoin pigment in liver and spleen at 60× magnification, respectively. (**P**) Histological score is based on liver inflammation and hemozoin deposition in the liver and spleen. Data represented as mean ± SEM. *P < 0.05, **P < 0.01, ***P < 0.001.

Acute anemia triggers compensatory stress erythropoiesis to increase the production of RBCs. However, the RBCs produced in this manner are often released into the bloodstream before they are fully mature. These immature RBCs (alias reticulocytes) can be identified via their surface expression of CD71 (2, 13). To confirm whether phlebotomy-induced anemia induces reticulocytosis, we examined the percentage of Ter119^+^CD71^+^ RBC (*aka* reticulocytes) in phlebotomized and control mice via flow cytometry. Fig. S1K demonstrates the gating strategy for reticulocyte enumeration. As anticipated, phlebotomized mice exhibited approximately 1.7-fold greater abundance of reticulocytes compared to controls (Fig. 1A and B), indicating phlebotomy-induced anemia leads to an increase in the percentage of circulating reticulocytes.

Anemia is a known sequela of malaria, but whether pre-existing anemia could drive the severity of the disease is not completely understood. To demonstrate that anemia can exacerbate malaria, we infected both control and phlebotomized male mice with *P. yoelii*. Anemic mice showed a sharp decline in body weight post-infection (p*.*i.) that correlated with significant increases in parasitemia over the span of days 4–6 p*.*i. (Fig. 1C through E). Non-anemic mice initially exhibited similar loss in body weight up to day 4, but recovered thereafter (Fig. 1C), associated with only a modest rise in parasitemia (Fig. 1D and E). The anemic *P. yoelii-*infected mice also showed a decrease in liver and spleen weight (Fig. 1F and G) compared to their non-anemic counterparts. Additionally, the serum isolated from the anemic parasitized mice had a stark yellowish appearance, which may be a consequence of hemolysis and/or malarial hepatitis and accumulation of bilirubin (Fig. 1H). Furthermore, fluorescence microscopy analysis of blood smears collected on day 6 p.i. revealed that anemic mice exhibited a greater proportion of parasitized RBC (Fig. 1I).

Next, we sought to assess the correlation between parasitemia and inflammation by measuring the inflammatory mediators in the sera collected on day 6 p.i. Both anemic and non-anemic mice infected with *P. yoelii* exhibited significantly elevated levels of the acute phase proteins Lcn2 and SAA (Fig. 1J and K), as well as the chemokine keratinocyte chemoattractant (KC or CXCL1) (Fig. S2D). Notably, Lcn2 and SAA levels were most prominently increased in anemic *P. yoelii*-infected mice (Fig. 1J and K). The level of granulocyte colony-stimulating factor (GCSF, a growth factor promoting granulopoiesis) was moderately increased in the anemic *P. yoelii*-infected mice (Fig. S2E). On the other hand, while serum erythropoietin (EPO; the hormone that stimulates erythropoiesis) was elevated significantly p*.*i. in both groups, the increase was modest in the non-anemic mice but striking in the anemic mice (Fig. 1L). The greater increase in the latter may reflect a compensatory increase in RBC production in response to the anemic effect exerted additively by both phlebotomy and *P. yoelii* infection. These results were confirmed in two independent experiments (Fig. S3A through N). Similar results were also observed in female mice subjected to phlebotomy-induced anemia followed by *P. yoelii* infection (Fig. S4A through O). Taken together, these results affirmed that phlebotomy-induced anemia in mice can exacerbate malaria severity, irrespective of sex differences.

### *P. yoelii*-infected anemic mice suffered from severe hepatic injury and inflammation

The extent of liver damage has been shown to correlate with the severity of *Plasmodium* infection (14, 15). Accordingly, we sought to use the metrics of hepatitis markers to assess malaria severity among mice with and without phlebotomy-induced anemia. While all *P. yoelii*-infected mice showed elevated levels of liver injury markers such as ALT and TBA, these elevations were greater among mice with phlebotomy-induced anemia (Fig. S2A and B). Likewise, the level of the multi-organ injury marker AST was also significantly greater in anemic *P. yoelii*-infected mice (Fig. S2C). Hematoxylin-eosin (H&E)-stained liver sections revealed substantial accumulation of hemozoin (a brown crystalline pigment produced by malaria parasites from the degradation of hemoglobin) (16) and immune cell infiltration in the liver of anemic *P. yoelii*-infected mice compared to their non-anemic counterparts (Fig. 1M and P). Additionally, hemozoin in the spleen was more evident in anemic mice (Fig. 1N and P). Thus, our results indicate that pre-existing anemia not only increases parasitemia but also exacerbates the liver pathology associated with *P. yoelii* infection.

### Hemolytic anemia increased reticulocytes in mice

Next, we asked whether our findings could be recapitulated in a different model of anemia induced via hemolytic destruction of RBC by the chemical agent PHZ (17). Mice were injected with either a single dose of PHZ at various concentrations (10, 20, and 60 mg/kg b.w.), a double dose of PHZ at 60 mg/kg b.w., or phosphate-buffered saline (PBS). Blood samples were collected 48 h post-administration from mice that received a single dose and 24 h for the double dose. CBC results indicate that RBC, hematocrit, and MCV decreased proportionally with increasing concentration of PHZ (Fig. S5A through C). Interestingly, mice treated with a double dose of 60 mg/kg b.w. PHZ exhibited a significant increase in WBC, whereas a single dose notably decreased these cell populations (Fig. S5D). Circulating neutrophils and monocytes increased with both single and double doses of 60 mg/kg b.w. PHZ (Fig. S5E and F). Furthermore, a single dose of 60 mg/kg b.w. PHZ reduced lymphocytes, while a double dose increased these cells (Fig. S5G). The post-mortem examination at 48 h after PHZ injection revealed significant splenic blackening and enlargement in mice that received 20 or 60 mg/kg (single or double dose, Fig. S5H and I). The serum samples from mice treated with a double dose of 60 mg/kg PHZ exhibited a reddish-brown discoloration, indicative of pronounced hemolysis (Fig. S5J), and were therefore excluded from subsequent experiments.

**Fig 2:**
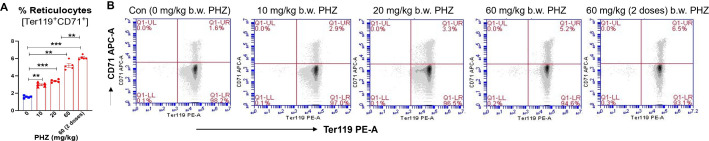
PHZ-induced hemolytic anemia increased reticulocytes. PHZ (0, 10, 20 mg/kg [one dose] and 60 mg/kg [one and/or two doses]) was administered i*.*p. to female WT mice (10-week-old, *n* = 5/group). Forty-eight hours after PHZ administration, blood samples were collected for reticulocyte analysis (Ter119^+^CD71^+^ cells). (**A**) % Reticulocytes. (**B**) Representative dot plots for reticulocytes. Data represented as mean ± SEM. *P < 0.05, **P < 0.01, ***P < 0.001.

PHZ treatment has been previously shown to induce reticulocytosis that peaks after 2–4 days (12, 18, 19). To determine the dose-response effect of PHZ on reticulocytosis, we collected blood at 48 h post-PHZ treatment and assessed the abundance of circulating Ter119^+^CD71^+^ reticulocytes. A proportional increase in circulatory reticulocytes was observed at 0, 10, 20, 60 (single dose), and 60 (double dose) mg/kg b.w. of PHZ (Fig. 2A and B). These results were confirmed in two separate experimental groups in females, as shown in Fig. S6A and B.

### PHZ-induced anemic mice exhibited heightened *Plasmodium* infection

**Fig 3:**
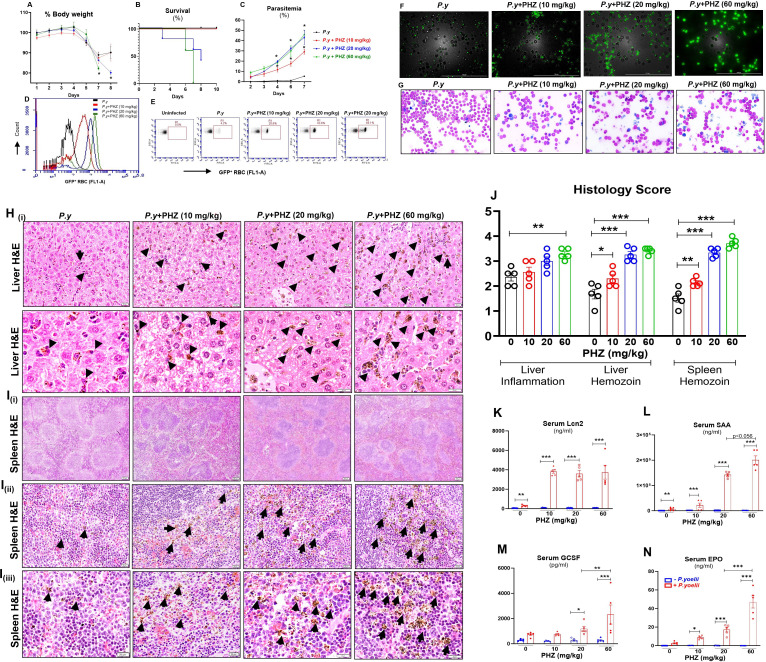
PHZ-induced anemic mice exhibited more severe *P. yoelii* infection. WT control mice and PHZ-induced (0, 10, 20, and 60 mg/kg, i*.*p. single dose) anemic mice (10-week-old females, *n* = 5/group) were infected with *P. yoelii* 48 h post-PHZ treatment and euthanized on days 7–8. (**A**) % Body weight. (**B**) Survival graph. (**C**) % Parasitemia. (**D**) Flow cytometry representation of % GFP-*P. yoelii*-infected RBC in histogram and (**E**) dot plots at day 7 p.i. (**F**) Representative images of GFP-*P. yoelii*-infected RBC visualized under a fluorescence microscope. (**G**) Representative images of infected RBC (Giemsa staining). Scale bar = 100 µm; magnification = 40× (bright field and FITC). (**H**) Liver histology depicted at 20× and 40× magnification; bars are 20 µm. (**I**) Spleen histology depicted at (I_i_) 4×, (I_ii_) 20×, and (I_iii_) 40×; bars are (I_i_) 200 µm and (I_ii_, I_iii_) 20 µm. PRBCs and hemozoin pigments are marked with black arrows. (**J**) Histological scores are based on liver inflammation and hemozoin deposition in the liver and spleen from *P. yoelii*-infected mice. Serum samples were analyzed for cytokines, (**K**) Lcn2, (**L**) SAA, (**M**) GCSF, and (**N**) EPO, measured by ELISA. Data represented as mean ± SEM. *P < 0.05, **P < 0.01, ***P < 0.001.

To evaluate the impact of PHZ-induced hemolytic anemia on *P. yoelii* infection, female mice were administered varying doses of PHZ (10, 20, and 60 mg/kg b.w.) or PBS. Each group was divided into two subgroups: one was infected with *P. yoelii* and the other received PBS. A rapid decrease in body weights across all groups was observed starting on day 4 p.i*. P. yoelii*-infected PHZ-induced anemic mice exhibited significant body weight loss 6–7 days p*.*i., especially in the 20 and 60 mg/kg groups. In contrast, the mice received 0 and 10 mg/kg b.w. PHZ began to regain body weight after day 7 (Fig. 3A). Importantly, the percent survival of *P. yoelii*-infected PHZ 60 mg/kg < PHZ 20 mg/kg < PHZ 10 mg/kg = control *P*. *yoelii*-infected groups (Fig. 3B). PHZ-induced anemic mice showed an increase in parasitemia from day 3 p.i. compared to non-anemic *P. yoelii*-infected mice (Fig. 3C). Notably, a positive correlation was observed between PHZ concentration and parasitemia (Fig. 3C through E). Post-mortem (7–8 days p*.*i.) organ weights showed significant loss of liver, spleen, and lung mass in mice that received both 20 and 60 mg/kg PHZ (Fig. S7A through C). Moreover, Giemsa staining and fluorescent microscopy revealed a dose-dependent correlation, with severity of anemia associated with more extensive infected RBC on 7–8 days p*.*i. (Fig. 3F and G). We have validated these results in two separate experimental groups in females, as shown in Fig. S8A through H. To rule out sex-based variability, we induced hemolytic anemia with PHZ in male mice and confirmed that they also exhibited severe *P. yoelii* infection (Fig. S9), similar to the females.

### PHZ-induced anemic *P. yoelii*-infected mice exhibited heightened liver injury markers and elevated liver and spleen inflammation

To assess liver injury in PHZ-induced anemic *P. yoelii*-infected mice, the principal serum markers of liver injury were measured. TBA, ALT, and AST levels were significantly elevated in mice upon infection (Fig. S7D through F), indicating severe hepatitis and multi-organ damage in PHZ-treated *P. yoelii*-infected mice. However, serum cholesterol levels were significantly reduced in all *P. yoelii*-infected groups (Fig. S7G), suggesting interrupted hepatic cholesterol biosynthesis (20). The results indicate that the degree of liver injury is positively associated with the degree of PHZ treatment and the severity of infection.

Microscopic analysis of liver sections from 7 to 8 days p*.*i. revealed a significant accumulation of hemozoin in PHZ-induced anemic mice. The degree of accumulation of hemozoin (black arrows) and immune cell infiltration in parasitized mice was in the order of PHZ 60 mg/kg–PHZ 20 mg/kg > PHZ 10 mg/kg > control *P*. *yoelii*-infected group (Fig. 3H and J). Histological assessment of the spleen on day 7 p.i. showed loss of central germinal structure (Fig. 3Ii) and enhanced accumulation of hemozoin in PHZ (60 and 20 mg/kg)-induced anemic mice compared with PHZ 10 mg/kg and control groups (Fig. 3I [i, ii, and iii]). Histopathological analysis (Fig. 3J) corroborated that *P. yoelii* infection induced severe liver and splenic inflammation in pre-existing anemic mice compared to non-anemic controls.

### PHZ-induced anemic *P. yoelii*-infected mice showed a stark increase in inflammatory responses

To determine the impact of the PHZ-induced anemia with *P. yoelii* infection on inflammation, serum levels of various proinflammatory cytokines and general inflammatory markers were assessed at baseline and on days 7–8 p*.*i. Cytokines related to acute-phase responses (Lcn2 and SAA), neutrophil chemoattraction (KC), Th1-type responses (IFN-γ), regulatory immune responses (TNF-α), and erythropoiesis or hematopoiesis (IL-17, GCSF, and EPO) were measured. The results showed a significant elevation in Lcn2 and SAA levels in all mice following *P. yoelii* infection, with a more pronounced increase in cytokine levels under anemic (Fig. 3K and L) conditions. Furthermore, there was a significant increase in serum levels of TNF-α, IL-17, KC, and IFN-γ in parasitized mice that received 60 mg/kg b.w. of PHZ (Fig. S7H through K). In addition, GCSF and EPO levels increased in mice that received either 20 or 60 mg/kg b.w. of PHZ upon infection (Fig. 3M and N). These results indicate that pre-existing hemolytic anemia aggravates the severity of inflammation during *P. yoelii* infection.

### Blood transfusion from healthy normal donor mice ameliorated *P. yoelii* infection in pre-existing anemic mice by reducing the reticulocytes

**Fig 4:**
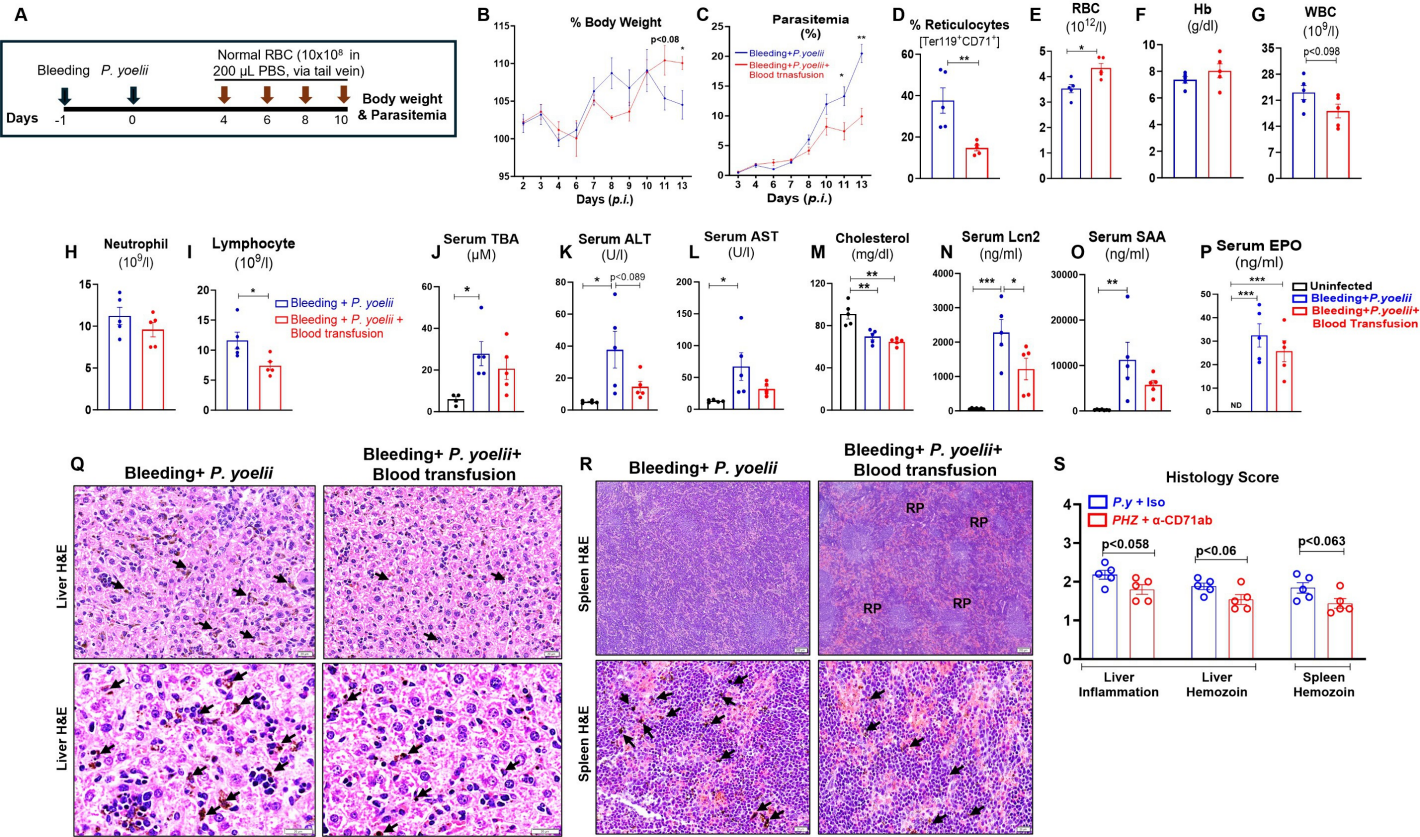
Blood transfusion reduced reticulocytes and ameliorated *P. yoelii* infection in phlebotomy-induced anemic mice. Phlebotomy-induced anemic mice (WT, 10-week-old females) were infected with *P. yoelii* 24 h after a blood draw of approximately 200 µL. The mice were divided into two groups (*n* = 5/group); one group received washed RBC (~10 × 10^8^ RBC resuspended in 200 µL of PBS) from healthy donor WT mice on days 4, 6, 8, and 10 p.i., and the next group received 200 µL of PBS at the same time points. The mice were euthanized on day 13 p.i. (**A**) Experiment design, (**B**) % body weight, (**C**) % parasitemia, (**D**) reticulocytes (Ter119^+^ CD71^+^ cells) analysis on day 13 p.i. CBC results for (**E**) RBC, (**F**) Hb, (**G**) WBC, (**H**) neutrophils, and (**I**) lymphocytes. Serum samples were collected from infected and uninfected control mice (WT, 10-week-old females, *n* = 5) and analyzed for (**J**) TBA, (**K**) ALT, (**L**) AST, and (**M**) cholesterol. Serum cytokines, (**N**) Lcn2, (**O**) SAA, and (**P**) EPO were measured by ELISA. (**Q**) Liver histology depicted at 20× and 40×: bars are 20 µm, (**R**) spleen histology depicted at 4× and 40×: bars are 200 and 20 µm, respectively. Hemozoin pigments are marked with black arrows. RP = red pulp. (**S**) Histological scores are based on liver inflammation and hemozoin deposition in the liver and spleen. Data represented as mean ± SEM. *P < 0.05, **P < 0.01, ***P < 0.001.

Blood transfusion is often performed to treat severe anemia (21). In our study, we confirmed that blood transfusion notably corrected anemia in phlebotomy-induced anemic mice (Fig. S10A through C), although no significant differences in immune cells were observed except for monocytes (Fig. S10D through G) in blood-transfused anemic mice. Clinical studies on this intervention generally concur that its application can enhance the survival rates in patients with severe malaria, presumably by mitigating anemia (21–24). How blood transfusion impacts *Plasmodium* infection itself, however, remains unclear. Given the profound impact of blood transfusion in human malaria, we proposed to perform a similar intervention in *P. yoelii*-infected mice with pre-existing anemia (induced via phlebotomy and PHZ treatment). We transfused RBC (10 × 10^8^ in 200 µL PBS, via the tail vein) from healthy normal donor mice into *P. yoelii*-infected anemic mice as illustrated in Fig. 4A; Fig. S11A. Blood transfusion protected *P. yoelii*-infected mice from further body weight deterioration (Fig. 4B; Fig. S11B) and reduced the percentage of parasitized RBCs (Fig. 4C; Fig. S11C). Blood transfused mice began to recover after day 10 p.i. as indicated by an increase in their body weights and a decrease in parasitized RBCs (Fig. 4B and C, Fig. S11B and C). Importantly, blood transfusion significantly reduced reticulocyte counts in *P. yoelii*-infected anemic mice (Fig. 4D; Fig. S11D), which likely contributed to their protection from *P. yoelii* infection. Conversely, hematologic analyses confirmed a significant increase in RBC counts (Fig. 4E; Fig. S11E) and an increase in hemoglobin (Fig. 4F; Fig. S11F) in blood-transfused *P. yoelii*-infected anemic mice compared to *P. yoelii*-infected anemic mice. There was also an increasing trend in hematocrit in blood-transfused *P. yoelii*-infected PHZ-induced anemic mice (Fig. S11G), but no differences were observed in MCV and MCH values (Fig. S11H and I). Furthermore, CBC results indicated a moderate reduction in the WBC and neutrophil counts, along with significantly reduced lymphocyte counts, in the blood-transfused *P. yoelii*-infected anemic mice (Fig. 4G through I). Moreover, cholestatic and inflammatory markers were reduced in blood transfused *P. yoelii*-infected groups compared to *P. yoelii*-infected anemic mice (Fig. 4J through L; Fig. S11K). Moreover, we observed marginal differences in cholesterol levels in blood transfused parasitized groups (Fig. 4M; Fig. S11L). Inflammatory markers, Lcn2 and SAA, decreased in blood-transfused *P. yoelii*-infected anemic mice (Fig. 4N and O). Additionally, serum EPO levels were significantly reduced in blood-transfused *P. yoelii*-infected mice, indicating that these mice were recovering from erythropoiesis (Fig. 4P; Fig. S11J). Histological analysis of liver and spleen sections showed a marginal reduction in the accumulation of hemozoin in blood-transfused *P. yoelii*-infected mice (Fig. 4Q and R); however, we noticed more red pulp in blood-transfused *P. yoelii*-infected spleen sections (Fig. 4R). These findings were further confirmed in two additional experimental groups in bleeding and PHZ-induced anemic mice, as presented in Fig. S12A through N. These results suggested that blood transfusion is a beneficial intervention in reducing parasitemia and ameliorating the effects of *P. yoelii* infection in anemic conditions, likely through mechanisms involving reticulocyte reduction and restoration of key hematologic parameters.

### Depletion of CD71^+^ reticulocytes protected mice from *P. yoelii* infection

**Fig 5:**
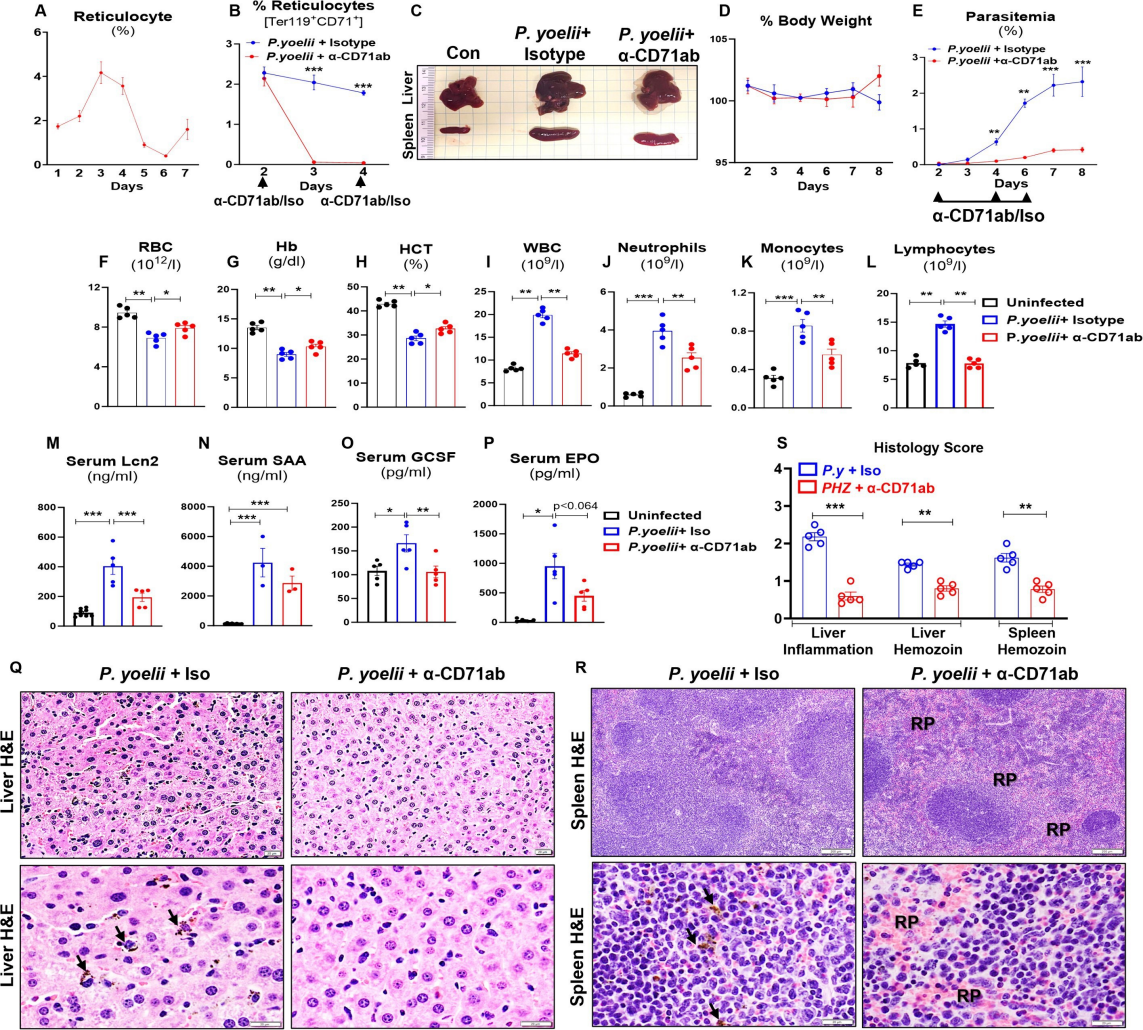
Depletion of CD71^+^ reticulocytes protected mice from *P. yoelii* infection. WT mice (10-week-old females) were infected with *P. yoelii* and then divided into two groups (*n* = 5/group); one group received anti-CD71 monoclonal antibody (α-CD71 mAb, 200 µg/mouse) on days 2, 4, and 6 p.i., and the other group received isotype IgG. The mice were euthanized on day 8 p.i. (**A**) % Reticulocytes during progression of *P. yoelii* infection. (**B**) % Reticulocytes in *P. yoelii+* isotype and *P. yoelii*+ α-CD71 mAb-treated groups. (**C**) Gross organ (liver and spleen) picture. (**D**) % Body weight. (**E**) % Parasitemia. Blood samples from uninfected mice and *P. yoelii*-infected mice that received either α-CD71 mAb or isotype were analyzed for CBC. Results for (**F**) RBC, (**G**) Hb, (**H**) HCT, (**I**) WBC, (**J**) neutrophils, (**K**) monocytes, and (**L**) lymphocytes. Serum cytokines, (**M**) Lcn2, (**N**) SAA, (**O**) GCSF, and (**P**) EPO were measured by ELISA. The liver and spleen sections were processed for histopathological changes. (**Q**) Liver histology depicted at 20× and 40× magnification: bars are 20 µm. (**R**) Spleen histology depicted at 4× and 40×: bars are 200 and 20 µm, respectively. PRBCs and hemozoin pigments are marked with black arrows. RP = red pulp. (**S**) Histological scores are based on liver inflammation and hemozoin deposition in the liver and spleen. Data represented as mean ± SEM. *P < 0.05, **P < 0.01, ***P < 0.001.

Reticulocytes are the primary targets for parasitization by many malarial parasites, including *P. vivax* and *P. yoelii*. Notably, *P. yoelii* demonstrates a 2.5-fold preference for reticulocytes over normocytes (2). One distinctive feature of reticulocytes is that they highly and selectively express CD71 (alias transferrin receptor 1) and would shed CD71 upon maturation (25). Hence, we sought to investigate whether anti-CD71 monoclonal antibody (α-CD71 mAb) could be leveraged to deplete circulating CD71^+^ reticulocytes and confer protection against *P. yoelii* infection. First, we determined that, upon *P. yoelii* infection, the percentage of circulating reticulocytes in mice significantly increased on day 2 and reached its peak at days 3–4 at approximately twofold higher than baseline (Fig. 5A). Thus, we opted to administer *P. yoelii*-infected mice with α-CD71 mAb starting on day 2, followed by repeated treatments on days 4 and 6 p.i. This intervention effectively depleted CD71^+^ reticulocytes, as confirmed by flow cytometry (Fig. 5B). The liver and spleen of *P. yoelii*-infected mice treated with α-CD71 mAb displayed gross reduction in dark coloration compared to those treated with isotype antibody (Fig. 5C). Although body weight differences were not initially observed, α-CD71 mAb-treated *P. yoelii*-infected mice began to regain weight on day 8 p.i. (Fig. 5D). Notably, α-CD71 mAb treatment protected *P. yoelii*-infected mice from the progressive increase in parasitemia (Fig. 5E). The anemia associated with *P. yoelii* infection, as indicated by the lowering of RBC counts, hemoglobin, and hematocrit p*.*i., was also partially alleviated by α-CD71 mAb treatment (Fig. 5F through H). Additionally, a substantial reduction in the WBC, neutrophil, monocyte, and lymphocyte counts was observed, indicating decreased systemic inflammation in the α-CD71 mAb-treated *P. yoelii*-infected mice (Fig. 5I through L). The inflammatory cytokines Lcn2, SAA, and GCSF decreased significantly in CD71^+^ reticulocyte-depleted *P. yoelii*-infected mice (Fig. 5M and O). Serum EPO levels also trended lower in α-CD71 mAb-treated *P. yoelii-*infected mice (Fig. 5P) in a manner reflecting the alleviation of malarial anemia (Fig. 5F through H).

The decreased gross hepatic and splenic darkening in the α-CD71-treated *P. yoelii*-infected mice compared to isotype-treated *P. yoelii*-infected mice (Fig. 5C) suggests that the protection was evident at the organ level as well. Our results indicated that α-CD71 mAb treatment significantly lowered both ALT and AST levels in *P. yoelii*-infected mice (Fig. S13A and B), affirming the alleviation of end-organ damage p*.*i. Serum cholesterol levels, however, remained unchanged between the α-CD71 mAb-treated and untreated groups (Fig. S13C). Histological examination of liver sections on day 8 p.i. revealed a significant reduction in the accumulation of hemozoin and immune cell infiltration in α-CD71 mAb-treated *P. yoelii*-infected mice (Fig. 5Q and S). Moreover, histological analysis of the spleen showed a loss of central germinal structures, red pulp (RP) integrity, and heightened hemozoin deposition in isotype-treated *P. yoelii*-infected mice (Fig. 5R and S). In contrast, these pathological features were significantly diminished in α-CD71 mAb-treated *P. yoelii*-infected mice (Fig. 5R and S). Collectively, our results underscore the significant impact of CD71^+^ reticulocyte depletion on the course of *P. yoelii* infection.

**Fig 6:**
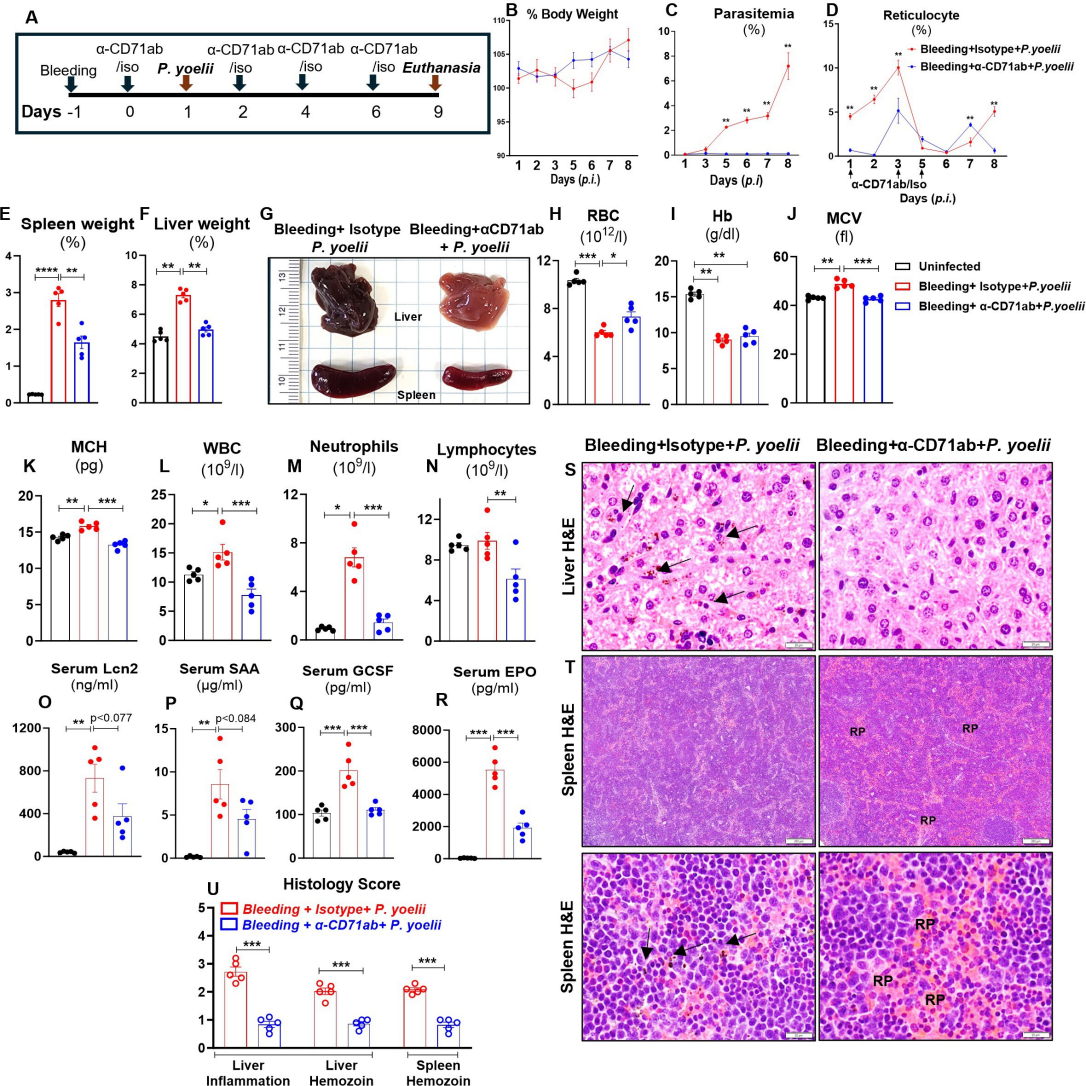
Depletion of CD71^+^ reticulocytes protected anemic mice from *P. yoelii* infection. Phlebotomy-induced anemic mice (10-week-old females, *n* = 5/group) received either α-CD71 mAb (200 µg/mouse) or isotype Ab 24 h post-bleeding. The mice were infected with *P. yoelii* 24 h after first α-CD71 mAb or isotype Ab administration. The mice were euthanized on day 9 p.i. (**A**) Overview of the study, (**B**) % body weight, (**C**) % parasitemia, (**D**) % reticulocytes (Ter119^+^CD71^+^ cells), (**E**) % spleen weight, (**F**) % liver weight, (**G**) gross organ (liver and spleen) picture. Blood samples were analyzed for CBC analysis. Results for (**H**) RBC, (**I**) Hb, (**J**) MCV, (**K**) MCH, (**L**) WBC, (**M**) neutrophils, and (**N**) lymphocytes. Serum samples were analyzed for cytokines, (**O**) Lcn2, (**P**) SAA, (**Q**) GCSF, and (**R**) EPO. (**S**) Liver histology depicted at 40× magnification: bars are 20 µm, (**T**) spleen histology depicted at 4× and 40× magnification: bars are 200 and 20 µm, respectively. PRBCs and hemozoin pigments are marked with black arrows. (**U**) Histological scores are based on liver inflammation and hemozoin deposition in the liver and spleen. Data represented as mean ± SEM. *P < 0.05, **P < 0.01, ***P < 0.001, ****P < 0.0001.

Next, we aimed to determine whether targeted depletion of CD71^+^ reticulocytes could also protect anemic mice from *P. yoelii* infection. To this end, we first induced anemia via phlebotomy, followed by α-CD71 mAb treatment and *P. yoelii* infection, as illustrated in the experimental plan (Fig. 6A). The subsequently observed body weights were comparable between α-CD71 mAb-treated and isotype antibody-treated *P. yoelii*-infected anemic mice (Fig. 6B). Nonetheless, the administration of α-CD71 mAb, compared to the isotype mAb, significantly protected anemic mice from *P. yoelii* infection. (Fig. 6C). In the isotype mAb-treated group, reticulocyte numbers initially increased on day 3 p.i., then decreased before increasing again after day 7 p.i. in a compensatory response to declining RBC numbers (Fig. 6D, red line). Notably, despite the substantially diminished parasite load in the α-CD71 mAb-treated mice, reticulocyte counts began to increase after 48 h of α-CD71 mAb treatment, reflecting stress erythropoiesis due to depletion of reticulocytes (Fig. 6D, blue line). Gross liver and spleen results confirmed that pre-anemic mice given α-CD71 mAb were protected from malaria-associated blackening of the liver and spleen, splenomegaly, and hepatomegaly (Fig. 6E through G). CBC results confirmed a significant increase in RBC, MCV, and MCH values in the α-CD71 mAb-treated *P. yoelii*-infected anemic mice, while hemoglobin remained comparable in both groups, suggesting recovery from anemia (Fig. 6H through K). Additionally, notable reductions in the WBC, neutrophil, and lymphocyte counts were observed, indicating decreased systemic inflammation in the α-CD71 mAb-treated *P. yoelii*-infected anemic mice (Fig. 6L through N). The systemic inflammatory markers Lcn2, SAA, and GCSF were also decreased significantly in α-CD71 mAb-treated *P. yoelii*-infected anemic mice (Fig. 6O through Q). Likewise, the serum EPO levels were significantly decreased in α-CD71 mAb-treated *P. yoelii-*infected anemic mice (Fig. 6R), highlighting the protective effect of α-CD71 mAb treatment against *P. yoelii* infection in anemic conditions. As was observed in the prior experiment, pre-anemic mice treated with α-CD71 mAb were protected from malaria-associated inflammation of the liver and spleen, splenomegaly, and hepatomegaly (Fig. 6E through G). Such protection was also evident at the histological level, i*.*e*.,* significantly reduced hemozoin accumulation in α-CD71 mAb-treated *P. yoelii*-infected mice (Fig. 6S through U) in both liver and spleen.

We have further validated these results in two separate experimental groups in females, as shown in Fig. S14A through N. Moreover, we also confirmed that PHZ-induced hemolytic anemic male mice treated with α-CD71 mAb were significantly protected against *P. yoelii* infection and anemia, as evident by the body weight, gross liver and spleen appearance, CBC results, inflammatory cytokines, and histological analysis (Fig. S15A through O). Taken together, these findings demonstrate the therapeutic potential of a targeted therapy against CD71^+^ reticulocyte in the non-anemic and pre-anemic context, for the treatment of *P. yoelii* infection.

## DISCUSSION

Reticulocytes, or immature RBCs, are the preferred target host cells for certain *Plasmodium* spp. that infect both humans and rodents (2, 13). For instance, human-specific *P. vivax* and the mouse-specific *P. yoelii* exhibit strong preferences for infecting reticulocytes (2). This selective tropism results from a complex interplay of host cell characteristics, parasite biology, and immune evasion mechanisms. Reports have demonstrated that reticulocytes provide several advantages to the parasites, including enhanced metabolic activity, increased protection from oxidative stress, and the ability to modify their membrane to better facilitate their intracellular expansion and immune evasion (2, 26). The circulating reticulocytes represent approximately 1%–2% of the total RBC population in the blood of a healthy individual. The tropism of *P. vivax* and *P. yoelii*, which are restricted to such a small RBC population in circulation, may explain, in part, their sub-lethal nature (2). On the other hand, *P. falciparum*, whose tropism is broader, is more likely to cause lethal infection (2). The importance of RBC tropism in determining malaria outcomes raises a prospective target that could curtail *Plasmodium* infection, thus alleviating malaria severity.

One prominent factor that can affect the proportion of circulating reticulocytes is anemia. Indeed, reticulocyte level is often used as a diagnostic marker for various types of anemia (2, 13). For instance, a low reticulocyte count is indicative of conditions such as pernicious anemia and iron deficiency anemia, whereas a high reticulocyte count suggests hemolytic anemia or anemia secondary to blood loss (2, 13). Taking into consideration the RBC tropism of *Plasmodium* spp., we reasoned that the anemia-associated increase in reticulocytes may aggravate *Plasmodium* infection. This may also be the case for the hypertensive BPH/2 mice, which, in a prior study, we found to display microcytic anemia, a greater reticulocyte population, and a more prolonged *P. yoelii* infection compared to the normotensive BPN/3 mice (27). To further determine the link between pre-anemic condition and malaria severity in this study, we employed phlebotomy and PHZ as tractable inducers of anemia in mice (12, 18, 28). Phlebotomy and PHZ treatment induced anemia and reticulocytosis in mice as anticipated, and more importantly, these anemic mice developed a more severe malarial pathology than control mice. Consistent with our observation, the study by *Zhu* et al. found that PHZ-induced anemia can accelerate *P. berghei*-induced cerebral malaria in mice (18). Though our study employed a different strain of mouse-specific *Plasmodium* spp., our findings concurred that pre-existing anemia could indeed pose a risk factor for severe malaria.

Epidemiologic data directly linking phlebotomy-induced or hemolytic anemia to malaria are currently lacking; however, such a link is apparent in the context of iron deficiency anemia. Intriguingly, iron deficiency anemia in humans is protective against malaria, and moreover, such protection could be negated by iron supplementation (29, 30). This is because, unlike hemolytic anemia, iron deficiency anemia presents defective reticulocytosis. It has been posited that the low reticulocyte count, rather than the anemia itself, is the primary factor that confers resistance to malaria (29, 30). Iron supplementation rectifies such defective reticulocytosis, thus increasing reticulocyte population and reinstating the susceptibility to malaria (29, 30). While the type of anemia may differentially affect the predisposition to malaria, the underpinning link between reticulocytosis and malaria susceptibility is well-conserved and consistent across the types of anemia studied thus far (18, 29, 30).

Another line of evidence in support of the anemia-malaria link can be gleaned from the use of blood transfusion in clinical practice to manage severe malarial anemia (21–24). Clinical reports generally agree that blood transfusion is beneficial in improving the survival of patients with severe malaria. Previous studies on mouse models of malaria have also demonstrated that whole blood transfusion drastically increased survival rate and reduced parasitemia in mice infected with *P. chabaudi* and *P. berghei* (31–33). Likewise, our study indicates that blood transfusion effectively mitigated the disease severity of *P. yoelii*-infected mice with phlebotomy-induced anemia (Fig. 4) and PHZ-induced hemolytic anemia (Fig. S11 and S12). Mice that received blood transfusions exhibited more weight gain, particularly following the third transfusion, and displayed reduced parasitemia. More importantly, blood transfusion normalized the reticulocyte levels that were elevated by phlebotomy and PHZ-induced hemolysis. It is plausible that such protection could be attributed, in large part, to the suppression of reticulocytosis post-transfusion, which effectively deprives *P. yoelii* of reticulocytes to parasitize. Notwithstanding its benefit, blood transfusion is often performed in clinical settings with the intent to alleviate malarial anemia, rather than to treat *Plasmodium* infection *per se*. A change in paradigm with respect to the latter may benefit patient care, especially if further studies can demonstrate the efficacy of blood transfusion as a first-line treatment rather than a delayed solution to malaria-induced anemia.

CD71 targeting therapy has been shown to be a promising anti-cancer treatment, since cancer cells overexpress CD71, i*.*e., a transferrin receptor, to sustain their high iron demand (34, 35). Intriguingly, a recent study found that the anti-cancer efficacy of α-CD71 mAb could also be mediated via selective depletion of a pro-tumorigenic subset of CD71^+^ reticulocytes (36). Such a notion raises the possibility that α-CD71 mAb could be repurposed as a treatment for malaria, given that many *Plasmodium* spp. exhibit a strong tropism toward CD71^+^ reticulocytes. *P. yoelii* (17XNL strain), for instance, prefers to invade CD71^+^ reticulocytes approximately 2.5 times more than erythrocytes (2). The mechanisms behind such tropism may involve the ability of *P. yoelii* to bind specific proteins or receptors on reticulocytes, potentially in a manner similar to how *P. vivax* (37, 38) and *P. berghei ANKA* (39) utilize CD71 as their entry receptor. Other potential explanations for such tropism include (i) reticulocytes may provide a nutrient-rich environment for parasite growth and replication (2), and (ii) reticulocytes exhibit greater membrane deformability than mature erythrocytes, which facilitates parasite invasion (40, 41). As anticipated, our results showed that α-CD71 mAb treatment in *P. yoelii*-infected mice substantially reduced parasitemia and provided protection against malarial hepatitis. The dose of α-CD71 mAb used herein was well-tolerated in mice, and intriguingly, it alleviates anemia (Fig. 6H through J; Fig. S14 and S15) rather than aggravating it. This could be likely attributable to the short duration of the α-CD71 mAb treatment, during which the benefit in impeding *P. yoelii* parasitization outweighs the transient disruption in stress erythropoiesis. Mechanistically, α-CD71 mAb is likely to confer protection by restricting *P. yoelii* access to its preferred host cell type and/or promoting the clearance of parasitized reticulocytes by immune cells. Thus, it is plausible that α-CD71 mAb treatment may be likewise effective against reticulocyte-restricted strains like *P. vivax* and *P. berghei ANKA*, but not against *P. falciparum* and *P. yoelii* YM (a virulent strain), which exhibit a broader RBC tropism (2, 42). Further studies are warranted to address these hypotheses and also to rule out the possibility of off-target or adverse effects.

**Fig 7:**
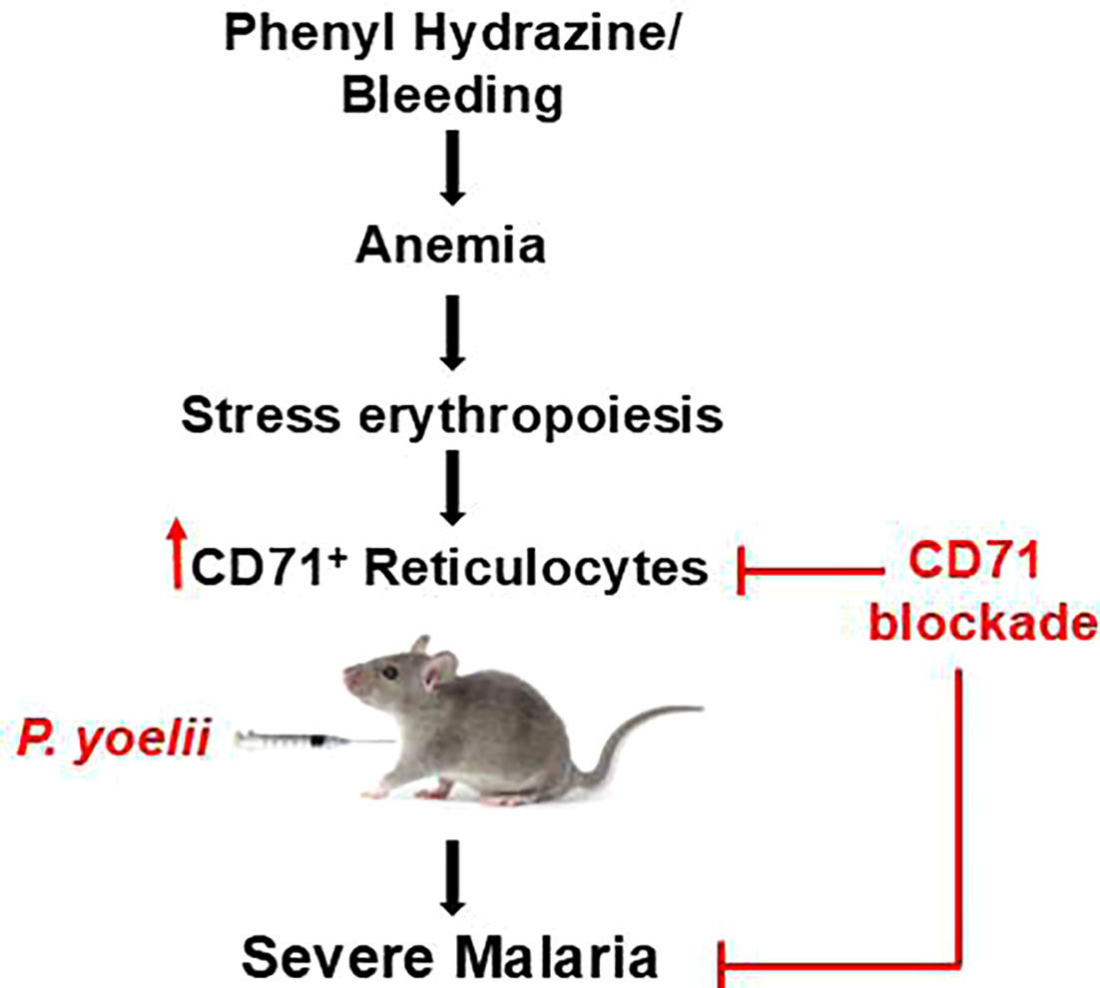
Pre-existing anemia promotes the expansion of reticulocytes, which contribute to increased parasitemia. Targeted depletion of CD71^+^ reticulocytes mitigated disease severity.

Taken together, our study delineates the interplay among anemia, reticulocytosis, and *P. yoelii* infection in mice (Fig. 7). CD71^+^ reticulocytes were identified as the primary determinant of disease outcomes, whereby (i) its elevation due to anemia increases predisposition for severe malaria, and (ii) its suppression upon blood transfusion alleviates malaria severity. To our knowledge, this study is the first to establish the proof-of-concept on the use of α-CD71 mAb as an immunotherapeutic for treating *Plasmodium* infection. Thus, we envision that α-CD71 mAb has the potential to be harnessed, alongside other targets involved in RBC parasitization (e*.*g., reticulocyte-binding 5 protein of *P. falciparum*) (43), as frontline medications for malaria. Future research should consider applying these findings to other *Plasmodium* species, including those causing human malaria and/or whose tropism is not restricted to only invading the reticulocytes. Investigating CD71-targeting approaches in human malaria could provide insights into novel therapeutic interventions, especially in cases of severe or life-threatening anemia.

## MATERIALS AND METHODS

### Mice

C57BL/6J wild-type mice (Stock # 000664) were procured from Jackson Laboratory and bred in-house in the Department of Laboratory Animal Resources, University of Toledo College of Medicine and Life Sciences. Mice were maintained under specific-pathogen-free conditions, housed in cages containing corn-cob bedding (Bed-O-Cob, The Andersons Co.) and nestlets (Cat# CABFM00088, Ancare Corp.), and fed *ad libitum* grain-based chow (LabDiet 5001). Mice were housed at 23°C with a 12 h light/dark cycle. Age- and sex-matched mice (males and females) were used for all the experiments. Animal handling was conducted according to the Institutional Animal Care and Use Committee-approved protocols.

### *Plasmodium yoelii* infection and quantification of parasitemia

The *P. yoelii* subsp. *yoelii*, strain 17XNL: PyGFP (catalog no. MRA-817) (44), which stably expresses the green fluorescent protein (GFP), was acquired from BEI Resources and kept as frozen stocks of parasitized RBC. Mice were infected with *P. yoelii*-parasitized RBC delivering 1 × 10^5^ parasites in 100 µL sterile PBS through intraperitoneal (i*.*p.) injections on each side of the abdomen. To assess parasitemia, whole blood from the tails of infected mice was repeatedly sampled (nick tail, 2 µL blood) over the course of infection and analyzed via flow cytometry. Parasitemia was defined as the percentage of GFP-positive RBCs in the whole blood. The parasitemia in control and anemic mice was also determined by microscopy on Giemsa-stained thin blood films. At the termination of the experiment, mice were euthanized via CO_2_ inhalation. The serum and organs were collected and stored at −80°C for further analysis.

### Phlebotomy-induced anemia

Phlebotomy-induced anemia was induced by submandibular phlebotomy of about 200 µL of blood from each mouse (*n* = 5) on two consecutive days (11, 12) in both male and female mice. Mice were weighed prior to each blood collection. After confirming anemia via CBC, mice were infected with *P. yoelii*.

### Phenylhydrazine-induced hemolytic anemia

Phenylhydrazine hydrochloride (PHZ; Sigma, MO, USA) was prepared as a 50 mg/mL stock solution in sterile PBS. Different concentrations of PHZ (10, 20, and 60 mg/kg b.w.) were diluted and administered via i*.*p. at 2 days prior to *P. yoelii* infection (17) in both male and female mice. The control group mice received the same amount of sterile PBS. For the dose-dependent PHZ study, mice were injected with either one or two doses of 60 mg/kg b.w. of PHZ. Mice in the control group received the same amount of PBS. The group receiving one dose of PHZ was infected with *P. yoelii* 2 days later. The group receiving two doses of PHZ was given the first dose 3 days before, and the second dose 1 day after being infected with *P. yoelii*. Hematological analyses were performed to confirm anemia (i*.*e., hemoglobin and hematocrit levels) prior to *P. yoelii* infection. Of note, PHZ induces the oxidation of hemoglobin, leading to its denaturation and precipitation, which results in the formation of fluorescent *“Heinz bodies*” within RBCs. This fluorescence, detectable by flow cytometry, can confound the accurate measurement of GFP^+^ RBC (*P. yoelii*-infected RBC), potentially leading to erroneous parasitemia readings (45, 46). To accurately estimate parasitemia in PHZ-treated mice, we subtract the readings (due to PHZ-associated RBC autofluorescence) of the uninfected subgroups from those of the infected groups. A dose-dependent increase in parasitemia was visually validated by preparing thin blood smears from both groups of mice and detecting GFP using FITC and Giemsa staining.

### Blood transfusion

Blood (~200 µL/day) was collected from C57BL/6J mice (6-week-old males) via submandibular bleeding to induce anemia. The mice were divided into two groups (*n* = 5/group). On days 2 and 3 post-phlebotomy, the first group received RBC (~10 × 10^8^ RBC resuspended in 200 µL sterile PBS and administered via tail vein injection), and the second group received only PBS. The donor blood was collected from mice that were age and sex-matched to the recipients. Different groups of mice, which had not been bled, were used as healthy donors for each day of blood transfusion. At 24 h after the second transfusion, blood samples were collected from the recipient mice for CBC analysis.

In another experiment, approximately 10 × 10^8^ RBC were resuspended in 200 µL of sterile PBS and injected into *P. yoelii*-infected mice (phlebotomy and PHZ-induced anemia) via the tail vein on days 4, 6, and 8 p*.*i. Percent parasitemia and TER119^+^ CD71^+^ reticulocytes were assessed using flow cytometry.

### *In vivo* neutralization of CD71^+^ reticulocytes

Ten-week-old *P. yoelii*-infected female mice were treated with either anti-CD71 antibody (α-CD71 mAb; 200 µg/mouse, InVivoMAb, BioxCell) or isotype IgG control (BioxCell) at days 2, 4, and 6 p.i. Percent parasitemia and reticulocytes were determined using flow cytometry.

A separate group of mice was subjected to phlebotomy-induced anemia (bled approximately 200 µL), and after 24 h, they were administered the first dose of α-CD71 mAb (200 µg/mouse) or isotype IgG control. After another 24 h, these mice were infected with *P. yoelii*. On days 2, 4, and 6 p.i., mice were given the second, third, and fourth doses, respectively, of either α-CD71 mAb or Isotype IgG control. Percent parasitemia and reticulocytes were assessed via flow cytometry.

In a different study, male mice were administered PHZ (60 mg/kg) to induce anemia. At 24 h post-PHZ treatment, mice were divided into three groups (*n* = 4/group): (i) PBS-treated, (ii) α-CD71 mAb (200 µg/mouse)-treated, and (iii) isotype-treated. The latter two groups were infected with *P. yoelii* 24 h after the first antibody administration. On days 2, 4, and 6 p.i*.,* mice were given the second, third, and fourth doses, respectively, of either α-CD71 mAb or Isotype IgG control. The mice were euthanized on day 9 p.i*.* due to the severity of diseases. Percent parasitemia and reticulocytes were assessed via flow cytometry.

### Flow cytometric analysis of reticulocytes

Blood (~2 µL) was collected via tail nick and suspended in 1 mL of sterile PBS (pH 7.0). Following centrifugation at 2,000 rpm for 5 min, the supernatant was removed, and cells were resuspended in 1 mL PBS containing 0.2% FBS. After two additional washes and centrifugation, cells were resuspended in 100 µL PBS + 0.2% FBS. Subsequently, cells were stained with fluorophore-conjugated anti-mouse monoclonal antibodies (Ter119-PE, CD71-APC, CD71-FITC, BD Biosciences) in staining buffer (PBS + 0.2% FBS) and incubated for 40 min at room temperature in the dark. Following a final wash, the stained cells were analyzed using the Accuri C6 flow cytometer (BD Biosciences) and BD Accuri C6 Software.

### Serum collection

Serum samples were collected at two distinct time points: the first, 2 weeks prior to the start of the experiment to allow recovery from anemia, and the second, at the time of euthanasia. Blood was collected via cardiac puncture in BD Microtainer tubes (BD Biosciences) and centrifuged at 10,000 rpm for 10 min. Hemolysis-free serum was carefully harvested and stored at −80°C until further analysis.

### Hematological analyses

On day 0, prior to *P. yoelii* infection, blood was collected in EDTA-coated microtubes (Sarstedt Inc.) for a CBC using an automated hematology analyzer VETSCAN HM5 Hematology Analyzer (ABAXIS HM5C & VS2, Allied Analytic). Parameters determined included WBC and RBC counts, hemoglobin concentration, hematocrit, MCV, MCH, and MCHC.

### Serum biochemical analyses

Serum TBA was measured using the Diazyme total bile acids assay kit (Cat # DZ042A) as per the manufacturer’s instructions. Furthermore, serum total cholesterol, AST, and ALT were measured using assay kits from Randox (Cat # CH200, AS101, and AL146, respectively) as per the manufacturer’s instructions.

### Serum ELISA

Serum lipocalin 2 (Lcn2, Cat # DY1857), serum amyloid A (SAA, Cat # DY2948-05), tumor necrosis factor alpha (TNF-α, Cat # DY410-05), interferon‐gamma (IFN-γ, Cat # DY485), interleukin 17 (IL-17, also known as IL-17A, Cat # DY5390), granulocyte colony-stimulating factor (GCSF, Cat # DY414), keratinocyte-derived chemoattractant (KC, alias CXCL1, Cat # DY453), mouse erythropoietin (EPO, Cat # DY959) were measured in hemolysis-free serum via DuoSet enzyme-linked immunosorbent assay (ELISA) kits from R&D Systems.

### Giemsa staining

Thin blood smears of uninfected and infected mice were fixed in methanol (Sigma-Aldrich) for 1–2 min and air dried. Next, the slides were submerged in Giemsa stain (1:20 in deionized water) (Sigma-Aldrich) for 15 min before they were rinsed in deionized water and air-dried for imaging and analysis using a Leica DM2500 LED optical microscope.

### Fluorescence microscopy

For fluorescence imaging, blood smears were fixed in methanol, imaged, and analyzed using a BioTek Cytation 5 Cell Imaging Multimode Reader (Agilent microscope). All images were captured at 40× magnification (bright field and FITC).

### Histology

Livers and spleen sections from uninfected and infected mice were fixed in 10% neutral buffered formalin, embedded in paraffin, sectioned (2 µm), and stained with H&E. Histological images were generated from the VS120 Virtual Slide Microscope (Olympus) and OlyVIA software. Histological scoring was conducted by a board-certified pathologist in a blinded fashion.

### Statistical analysis

Data are presented as mean ± SEM. Significance between two groups was assessed using Student’s *t*-test (unpaired, two-tailed), where *P* < 0.05 was deemed significant. For comparisons among means of three or more groups, one-way analysis of variance with pairwise multiple comparisons was employed. All statistical analyses were conducted using GraphPad Prism 9.0 software (GraphPad Software, Inc., La Jolla, CA).

## SUPPLEMENTAL MATERIAL

Supplemental material iai.00093-25-s0001.pdf Fig. S1 to S15.

ASM does not own the copyrights to Supplemental Material that may be linked to, or accessed through, an article. The authors have granted ASM a non-exclusive, world-wide license to publish the Supplemental Material files. Please contact the corresponding author directly for reuse.
