# Supplementary material for: Targeting anemia-induced CD71+ reticulocytes protects mice from Plasmodium infection
Source: Infect Immun. 2025 Jul 1;93(8):e00093-25. doi: 10.1128/iai.00093-25 (PMC12341372; doi:10.1128/iai.00093-25)
Supplement: Supplemental material — Fig. S1 to S15. [file iai.00093-25-s0001.pdf]

Supplementary Figure 1

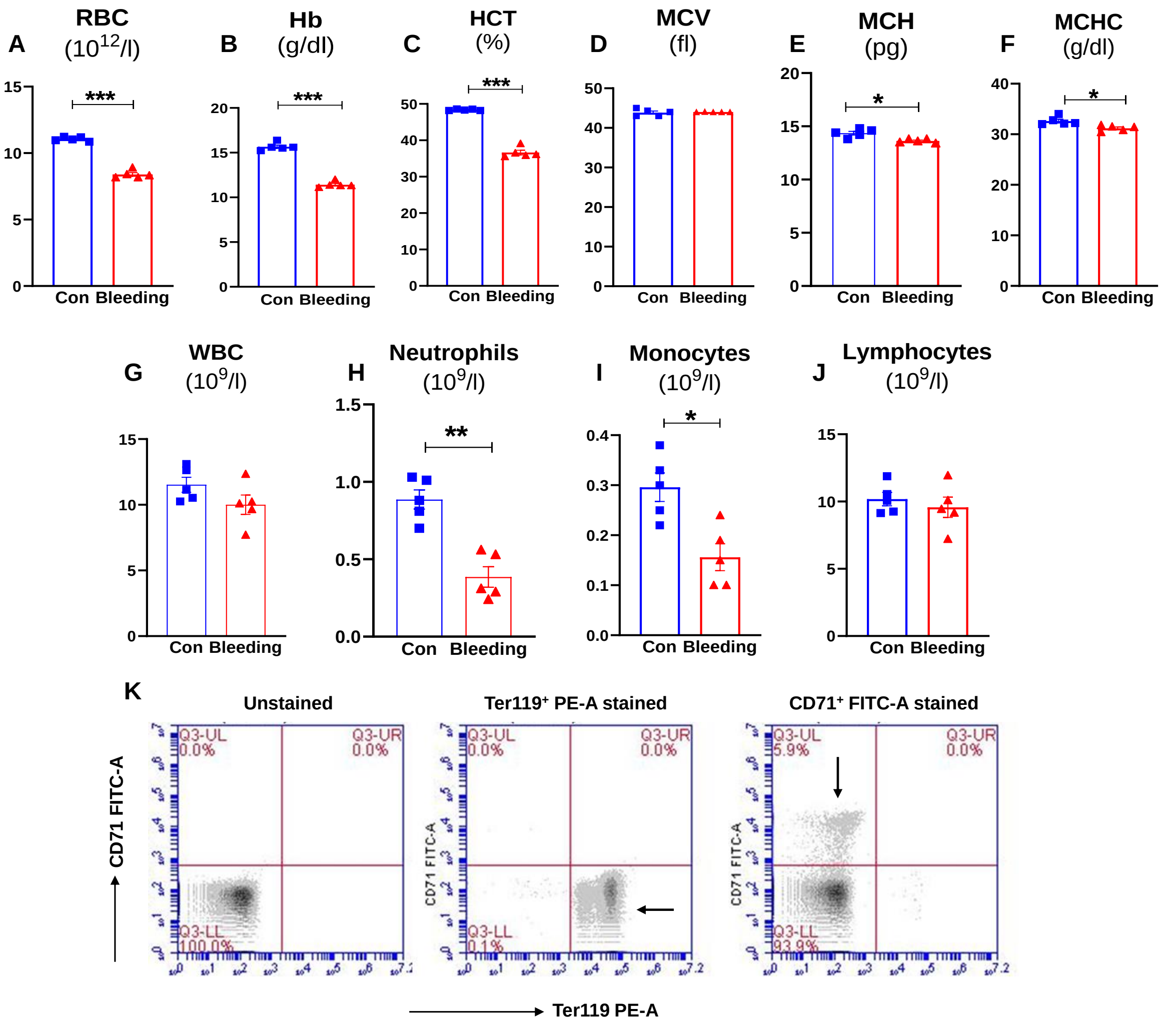

Supplementary Figure 1. Phlebotomy-induced anemia lowered RBC, hematocrit and hemoglobin count in mice.

Blood (~200 µL/ day) was collected from wild-type (WT) mice (10-week-old males) via submandibular bleeding on two consecutive days to induce anemia. Blood samples from WT control and phlebotomized mice (n=5/group) were collected in EDTA tubes 24 h after the second blood draw for complete blood count (CBC) analysis using the VetScan hematology analyzer. Results for: **(A)** Red blood cells (RBC); **(B)** Hemoglobin (Hb); **(C)** Hematocrit (HCT, the volume percentage of RBC in blood); **(D)** Mean corpuscular volume (MCV, average size of RBC); **(E)** Mean corpuscular hemoglobin (MCH average amount of hemoglobin in RBC); **(F)** Mean corpuscular hemoglobin concentration (MCHC, average concentration of hemoglobin in a given volume of RBC) via CBC. **(G)** White blood cells (WBC), **(H)** Neutrophils, **(I)** Monocytes, **(J)** Lymphocytes. Flow cytometry analysis for reticulocytes ($Ter119^+CD71^+$cells) in blood. Representative **(K)** gating strategy for single stained plots $Ter119^+$ and $CD71^+$ RBC. Data represented as mean ± SEM. *p<0.05, **p<0.01, ***p<0.001.

Supplementary Figure 2

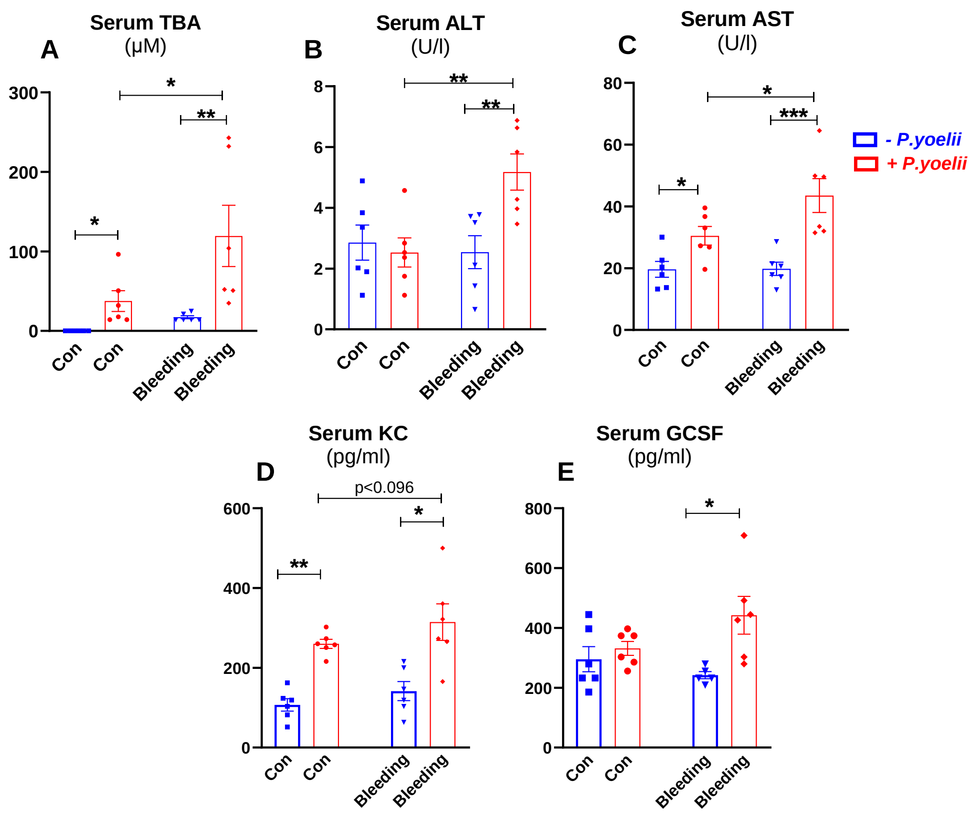

Supplementary Figure 2. *P. yoelii* infected anemic mice displayed stark inflammatory responses.

Serum samples were collected from WT control mice and phlebotomy-induced anemic mice (10-week-old males, n=6/ group) infected with *P. yoelii,* euthanized on day 6 *p.i.*, and analyzed for serum **(A)** total bile acids (TBA), **(B)** alanine transaminase (ALT), **(C)** aspartate aminotransferase (AST), and cytokines, (**D, E**) KC (CXCL 1), granulocyte colony-stimulating factor (GCSF) measured by ELISA. Data represented as mean ± SEM. *p<0.05, **p<0.01, ***p<0.001.

Supplementary Figure 3

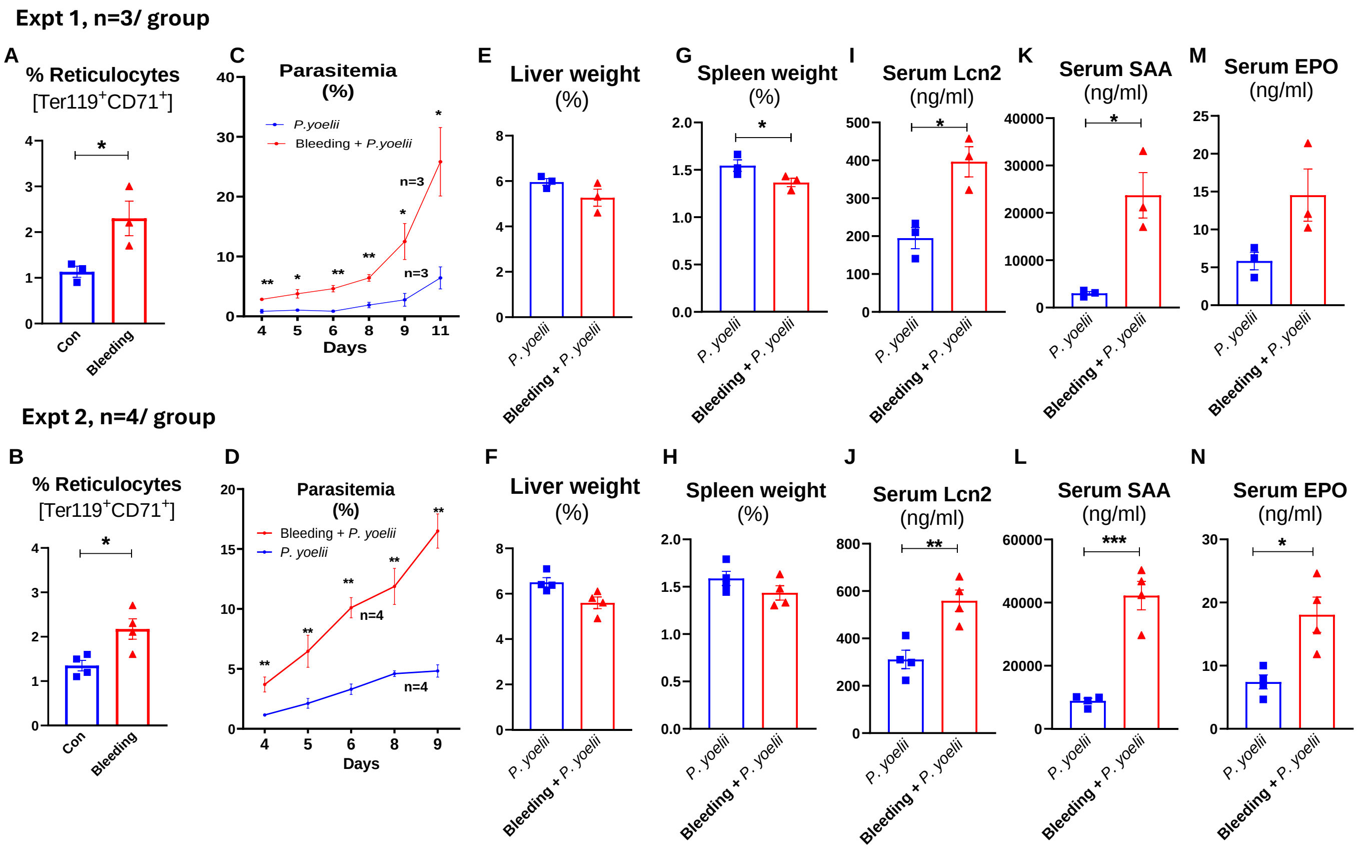

Supplementary Figure 3. Bleeding induced anemic mice had elevated reticulocytes and showed severe *P. yoelii* infection.

To induce anemia, blood was collected (~200 µL/day on two consecutive days) from 10-week-old male WT mice (n=3/group) for experiment 1 and (n=4/group) for experiment 2. Flow cytometry analysis was performed on the blood to assess reticulocytes (Ter119^{+}CD71^{+} cells) 24 h after the second blood draw. **(A, B)** % reticulocytes. Mice were infected with *P. yoelii* 24 h after the second blood draw. **(C, D)** % parasitemia, as indicated by GFP-positive RBC, was measured by flow cytometry during the infection. **(E, F)** % Liver weight, **(G, H)** % spleen weight. Serum samples were analyzed for cytokines: **(I, J)** lipocalin 2 (Lcn2), **(K, L)** serum amyloid A (SAA), and **(M, N)** erythropoietin (EPO) by ELISA. Data represented as mean ± SEM. *$p<0.05$, **$p<0.01$, ***$p<0.001$.

Supplementary Figure 4

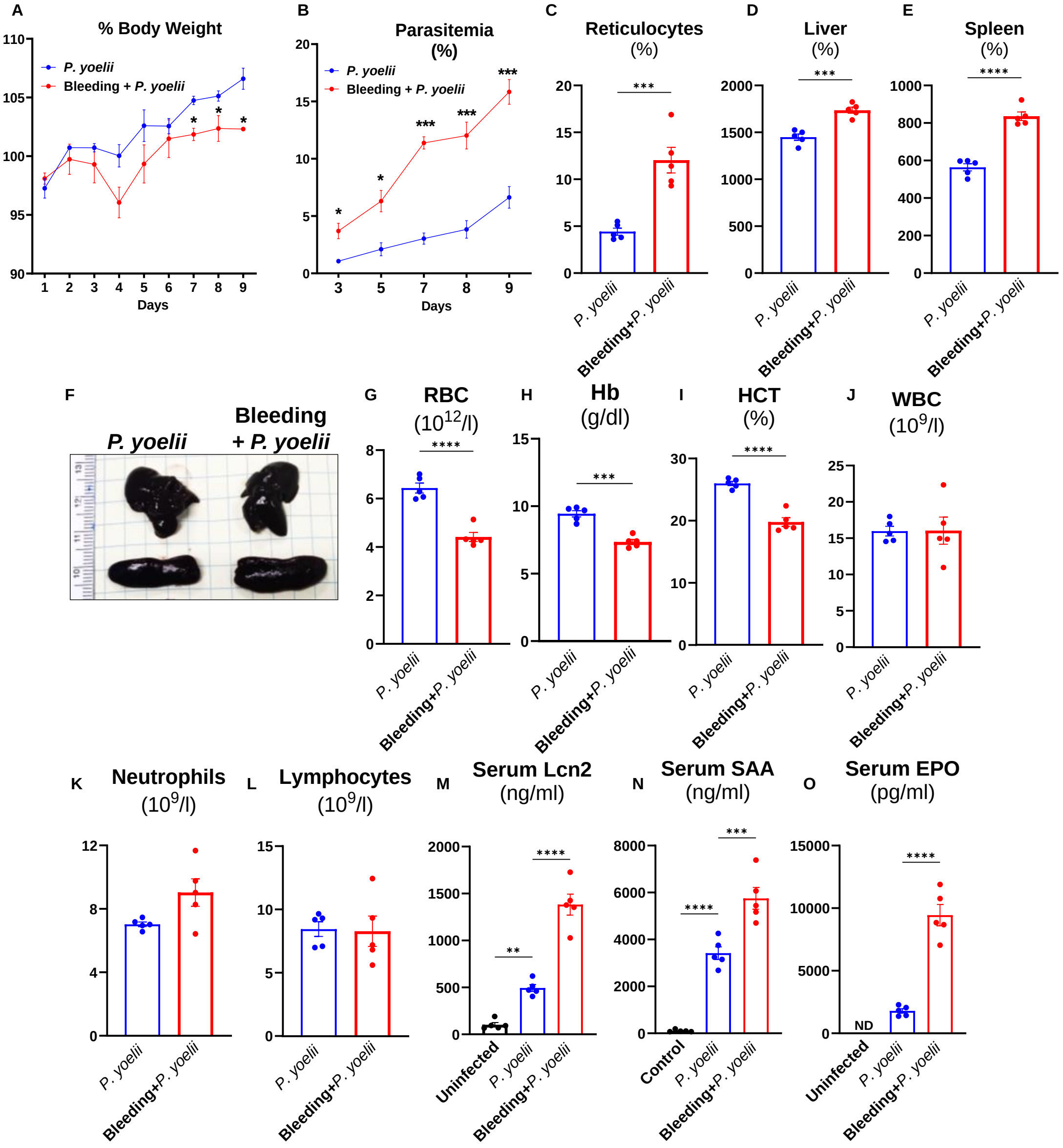

Supplementary Figure 4. Phlebotomy-induced anemic mice exhibited more severe *P. yoelii* infection.

Blood (~200 µL/day) was collected from 10-week-old female WT mice over two consecutive days via submandibular bleeding to induce anemia. Both anemic and non-anemic mice (n=5/group) were then infected with *P. yoelii* 24 h after the second blood draw and were euthanized on day 9 *p.i.* **(A)** % Body weight, **(B)** % parasitemia, **(C)** % Reticulocytes, **(D)** % liver weight, **(E)** % spleen weight, **(F)** Gross organ (liver and spleen) picture. Blood samples were analyzed for CBC analysis. Results for: **(G)** RBC, **(H)** Hb, **(I)** HCT, **(J)** WBC, **(K)** Neutrophils **(L)** Lymphocytes. Serum samples were analyzed for cytokines: **(M-O)** Lcn2, SAA, and EPO by ELISA. Data represented as mean ± SEM. *p<0.05, **p<0.01, ***p<0.001, ****p<0.0001.

Supplementary Figure 5

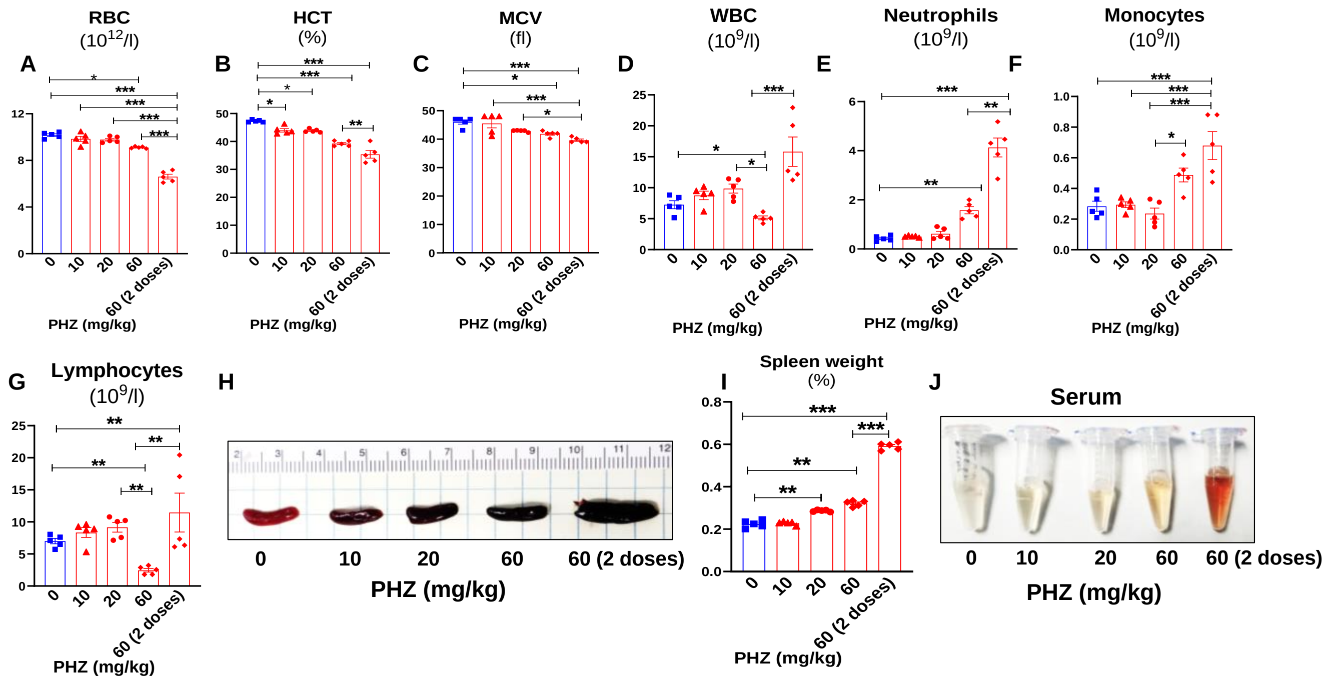

Supplementary Figure 5. Phenylhydrazine (PHZ) induced hemolytic anemia in mice.

PHZ [0, 10, 20 mg/kg (one dose), and 60 mg/kg (one and/or two doses)] was administered intraperitoneally (*i.p.*) to female WT mice (10-week-old). 48 h after PHZ administration, blood was collected for CBC analysis. Results for: **(A)** RBC, (B) HCT, **(C)** MCV, **(D)** WBC, **(E)** Neutrophils, **(F)** Monocytes, **(G)** Lymphocytes. **(H)** gross spleen picture, **(I)** % spleen weight, **(J)** serum color changes in a dose-dependent manner 48 h after PHZ treatment. Data represented as mean ± SEM. *p<0.05, **p<0.01, ***p<0.001.

Supplementary Figure 6

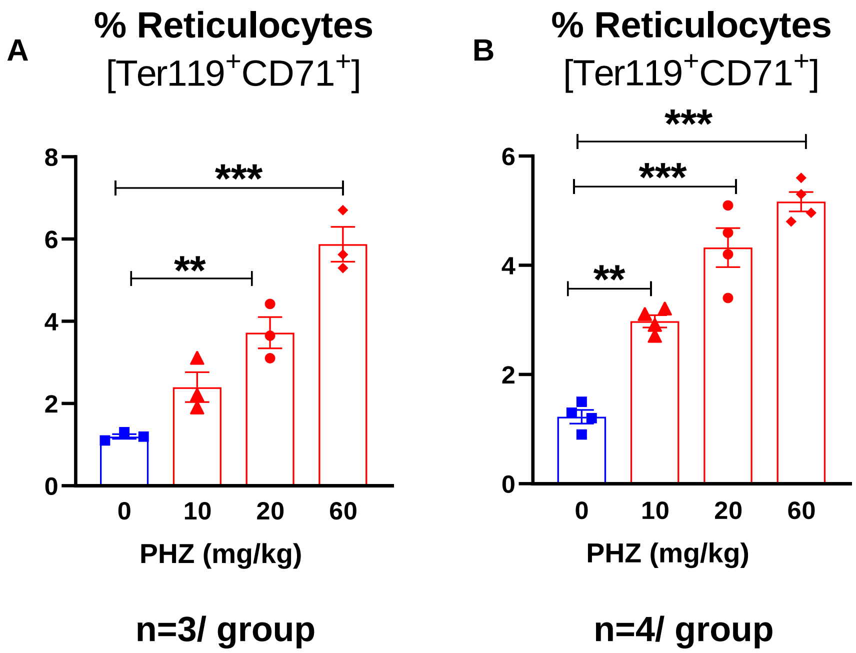

Supplementary Figure 6. PHZ induced hemolytic anemia elevated reticulocytes.

PHZ [0, 10, 20 and 60 mg/kg] was administered *i.p.* to female WT mice (10-week-old) for experiment 1 (n=3/ group) and experiment 2 (n=4/ group). 48 h after PHZ injection, blood samples were collected from WT control and PHZ-treated mice in EDTA tubes for reticulocyte analysis ($Ter119^{+}CD71^{+}$ cells) via flow cytometry. Bar graphs showing % reticulocytes for **(A)** Expt 1 and **(B)** Expt 2. Data represented as mean ± SEM. **p<0.01, ***p<0.001.

Supplementary Figure 7

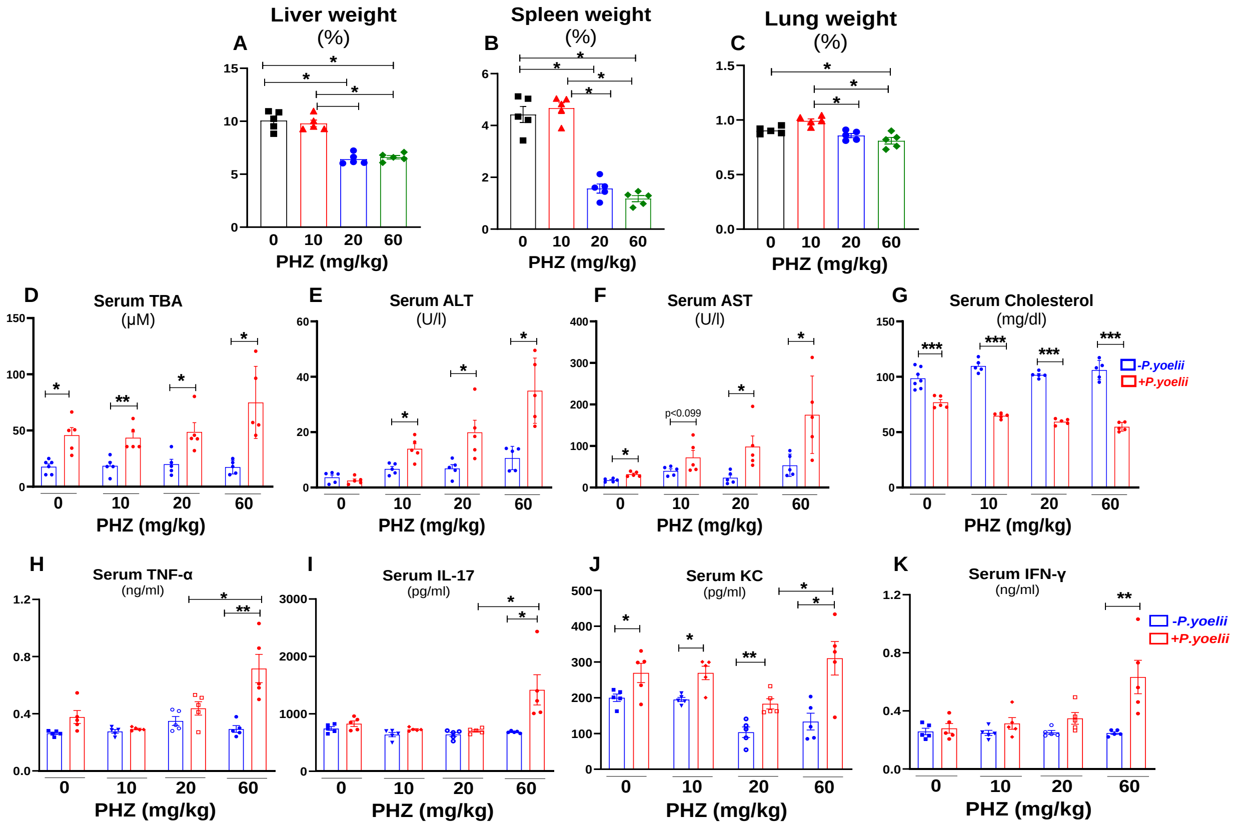

Supplementary Figure 7. PHZ-induced anemic mice displayed stark inflammatory responses.

WT control mice and PHZ-induced (0, 10, 20 and 60 mg/kg, *i.p.* single dose) anemic mice (10-week-old females, n=5/group) were infected with *P. yoelii* 48 h post-PHZ treatment. **(A)** % Liver weight, **(B)** % Spleen weight, **(C)** % Lung weight day 7-8 *p.i.*, Serum samples were analyzed for **(D)** TBA, **(E)** ALT, **(F)** AST and **(G)** cholesterol. Serum cytokines, **(H-K)** TNF-α, IL-17, KC (CXCL 1) and IFN- γ were measured by ELISA. Data represented as mean ± SEM. *p<0.05, **p<0.01, ***p<0.001.

Supplementary Figure 8

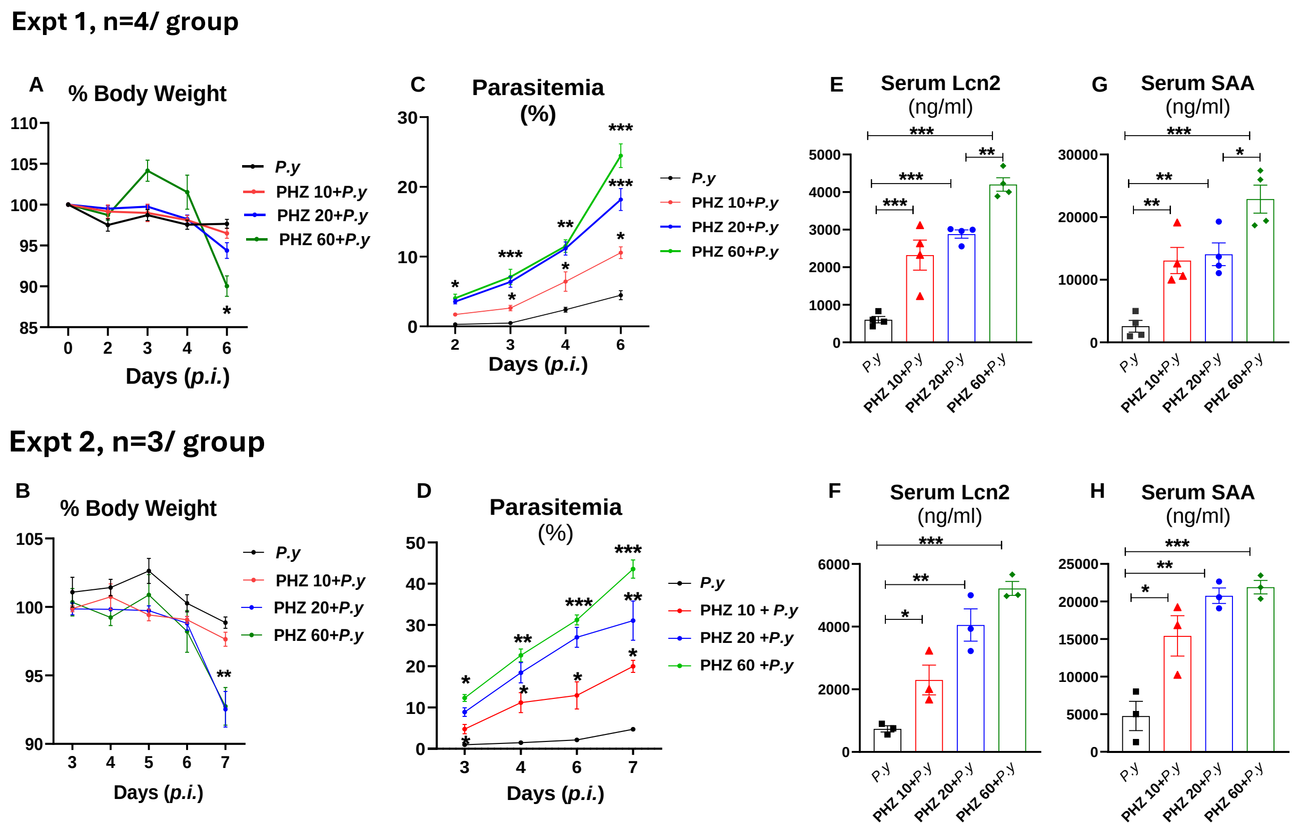

Supplementary Figure 8. PHZ-induced anemia showed severe *P. yoelii* infection.

WT control mice and PHZ-induced (0, 10, 20 and 60 mg/kg bw., *i.p.* single dose) anemic mice (10-week-old females) (experiment 1: n=4/group and experiment 2: n=3/group) were infected with *P. yoelii* 48 h post-PHZ treatment and euthanized on days 6-7. **(A, B)** % Body weight. **(C, D)** % Parasitemia. Serum samples were analyzed for cytokines after 6-7 days *p.i.*, **(E, F)** Lcn2, **(G, H)** SAA by ELISA. Data represented as mean ± SEM. *p<0.05, **p<0.01, ***p<0.001.

Supplementary Figure 9

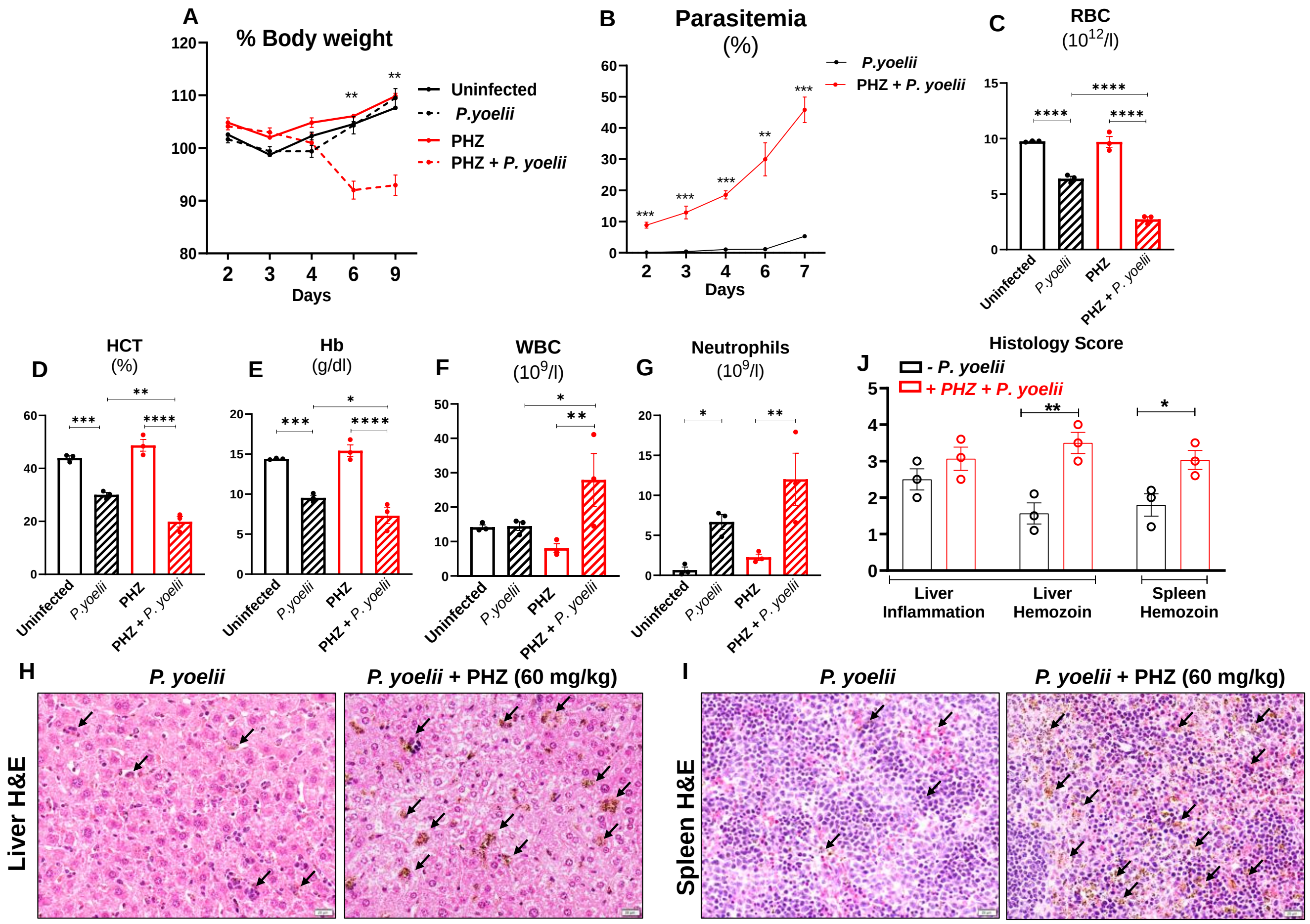

Supplementary Figure 9. PHZ-induced anemic mice exhibited severe *P. yoelii* infection.

WT control mice and PHZ-induced (60 mg/kg, *i.p.* single dose) anemic mice (10-week-old males, n=3/group) were infected with *P. yoelii* 48 h post-PHZ treatment and euthanized on day 9 *p.i.* **(A)** % Body weight. **(B)** % Parasitemia. Blood samples from infected and uninfected mice were analyzed for CBC. Results for: **(C)** RBC, **(D)** HCT, **(E)** Hb, **(F)** WBC, **(G)** Neutrophils. The liver and spleen sections were processed for histopathological changes. **(H)** Liver histology: Bars are 20 μm. **(I)** Spleen histology: Bars are 20 μm. Hemozoin pigments are marked with black arrows. **(J)** Histological score based on liver inflammation and hemozoin deposition in liver and spleen. Data represented as mean ± SEM. *p<0.05, **p<0.01, ***p<0.001, ****p<0.0001.

Supplementary Figure 10

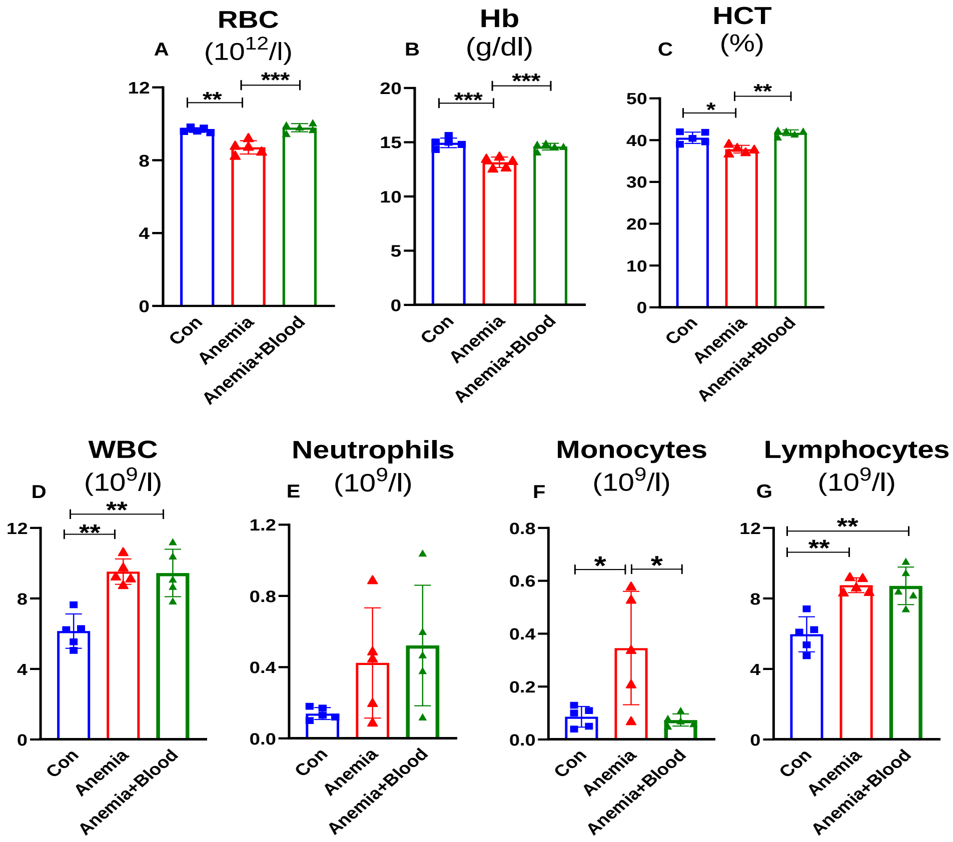

Supplementary Figure 10: Blood transfusion notably corrected anemia in phlebotomy-induced anemic mice.

Blood (~200 µL/ day) was collected from male WT mice (6-week-old) via submandibular bleeding to induce anemia. The mice were divided into two groups (n=5/group); one group received washed RBC (~10 × 10^8 RBC resuspended in 200 µL of PBS) from healthy donor WT mice on days 2 and 3 post-bleeding, next group received 200 µL of PBS at the same time points. 24 h after the second transfusion, blood samples were collected from both anemic and nonanemic (6-week-old WT males, n=5) mice for CBC analysis. Results for: **(A)** RBC, **(B)** Hb, **(C)** HCT, **(D)** WBC, **(E)** Neutrophils, **(F)** Monocytes, **(G)** Lymphocytes. Data represented as mean ± SEM. *$p<0.05$, **$p<0.01$, ***$p<0.001$.

Supplementary Figure 11

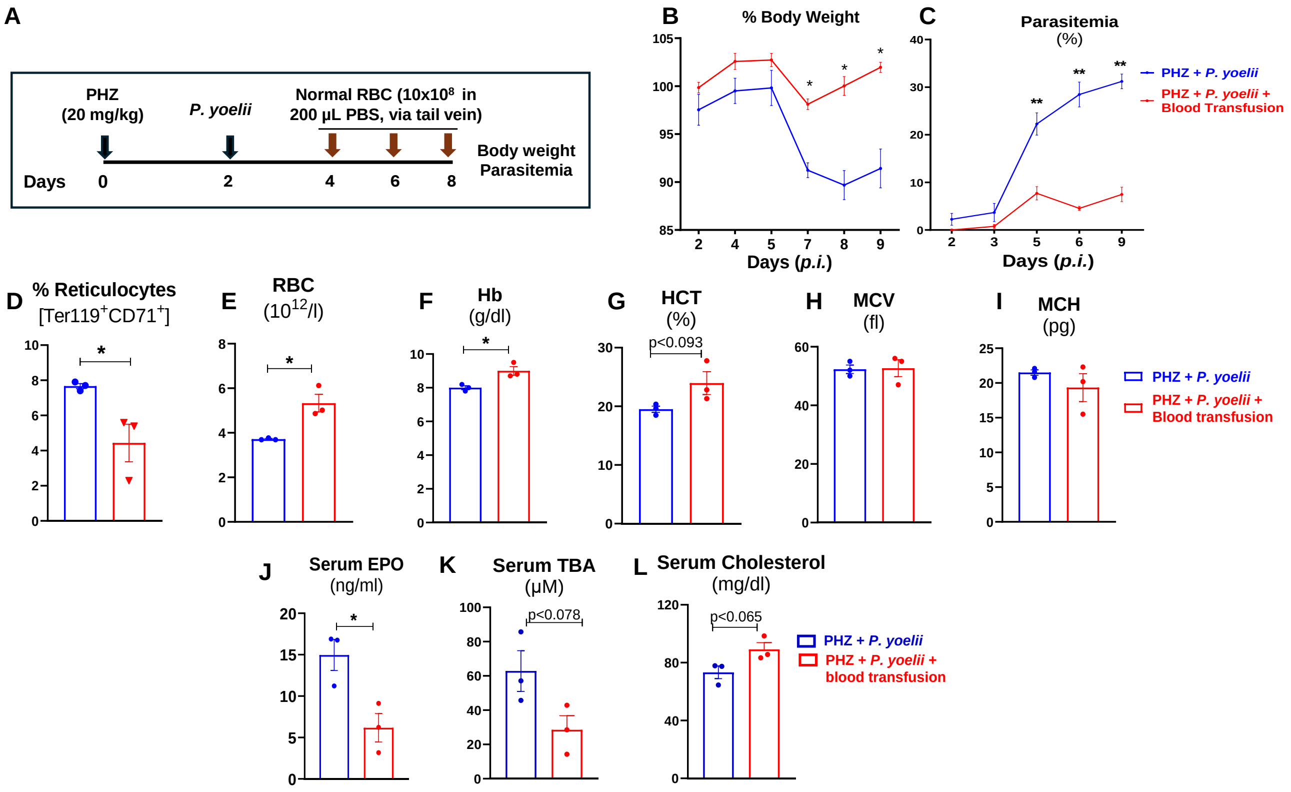

Supplementary Figure 11: Blood transfusion decreased reticulocytes in circulation and mitigate *P. yoelii* infection in PHZ-induced anemic mice.

PHZ-induced (20 mg/kg, *i.p.* single dose) anemic mice (10-week-old males) were infected with *P. yoelii* 48 h post-PHZ treatment. The mice were divided into two groups (n=3/group); one group received washed RBCs (~10 × 10^8 RBC were resuspended in 200 µL of PBS) from healthy donors WT mice on days 4, 6, 8 post-PHZ treatment, next group received 200 µL of PBS at the same time points. **(A)** Experiment design, **(B)** % Body weight, **(C)** % Parasitemia, **(D)** Reticulocytes analysis on day 9 *p.i.* Blood samples were analyzed for CBC. Results for: **(E)** RBCs, **(F)** Hb, **(G)** HCT, **(H)** MCV and **(I)** MCH. Serum samples were analyzed for **(J)** EPO, **(K)** TBA, **(L)** cholesterol. Data represented as mean ± SEM. *p<0.05 and **p<0.01.

Supplementary Figure 12

Expt 1, n=3/ group, bleeding induced anemia +*P. y* + blood transfusion

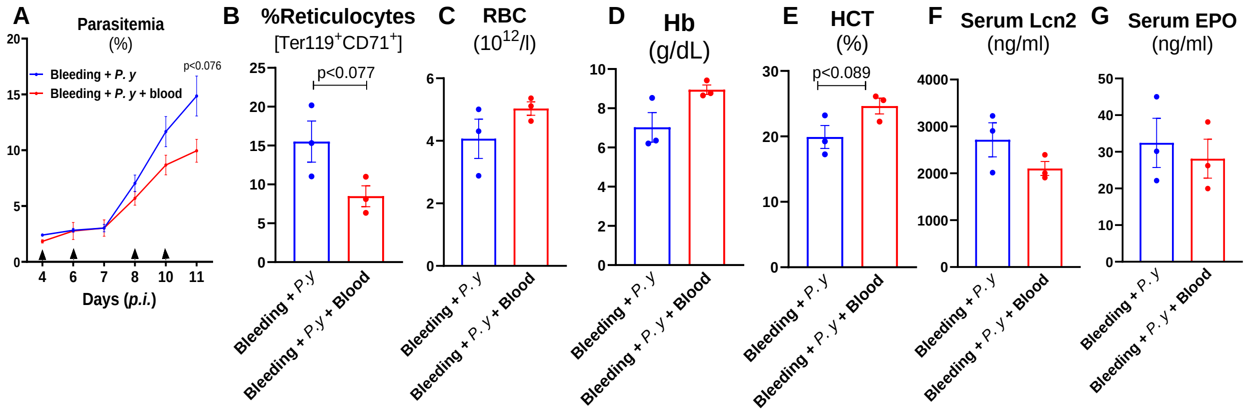

Expt 2, n=3/ group, PHZ induced anemia+ *P. y* + blood transfusion

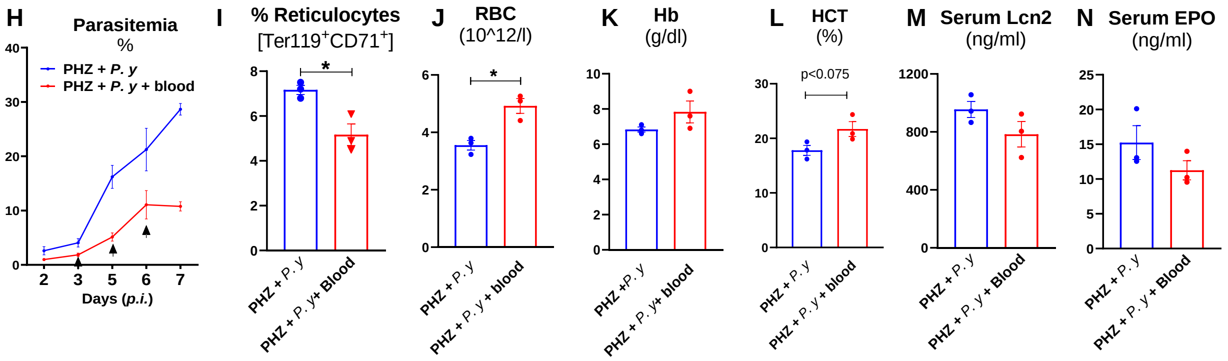

Supplementary Figure 12: RBC transfusion reduced reticulocytes and ameliorated *P. yoelii* infection.

In Experiment 1, bleeding-induced anemic mice (WT, 8-week-old females) were infected with *P. yoelii* 24 h after a blood draw of approximately 200 µl. The mice were divided to two groups (n=3/ group); one group received washed RBC (~10×10^8 RBC resuspended in 200 µL of PBS) from healthy donors WT mice on days 4, 6, 8, and 10 *p.i.*, next group received 200 µL of PBS at the same time points. The mice were euthanized on day 11 *p.i.* **(A)** % Parasitemia, **(B)** Reticulocytes ($Ter119^+$ $CD71^+$ cells) analysis on day 11 *p.i.* CBC results for: **(C)** RBC, **(D)** Hb, **(E)** HCT. Serum samples analyzed for cytokines **(F)** Lcn2 and **(G)** EPO by ELISA. Data represented as mean ± SEM.

In Experiment 2, PHZ-induced (20 mg/kg bw., *i.p.* single dose) anemic mice (8-week-old males) were infected with *P. yoelii* 48 h post-PHZ treatment. The mice were divided into two groups (n=3/ group); one group received washed RBCs (~10×10^8 RBC were resuspended in 200 µL of PBS) from healthy donors WT mice on days 3, 5, 6 post-PHZ treatment, next group received 200 µL of PBS at the same time points. **(H)** % Parasitemia, **(I)** Reticulocytes analysis on day 7 *p.i.* Blood samples were analyzed for CBC. Results for: **(J)** RBC, **(K)** Hb, **(L)** HCT. Serum samples were analyzed for **(M)** Lcn2 and **(N)** EPO. Data represented as mean ± SEM. *p<0.05.

Supplementary Figure 13

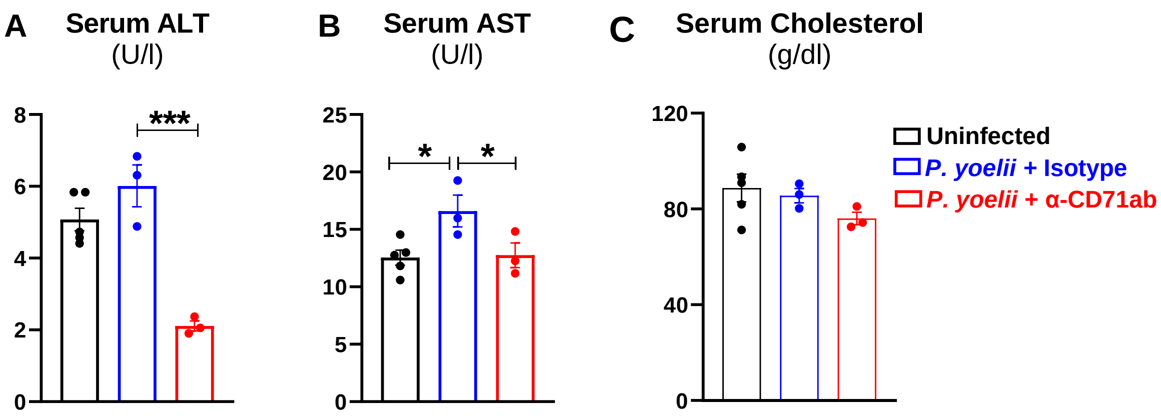

Supplementary Figure 13. α-CD71 mAb treatment diminished liver inflammation in *P. yoelii* infected mice.

WT mice (10-week-old females) were infected with *P. yoelii*. The mice were divided into two groups (n=3-5/group); one group received α-CD71 mAb (200 µg/mouse) on days 2, 4, and 6 *p.i.* and the other group received isotype IgG. Serum samples were collected on day 8 *p.i.* and analyzed for **(A)** ALT, **(B)** AST and **(C)** cholesterol. Data represented as mean ± SEM. *$p<0.05$ and ***$p<0.001$.

Supplementary Figure 14

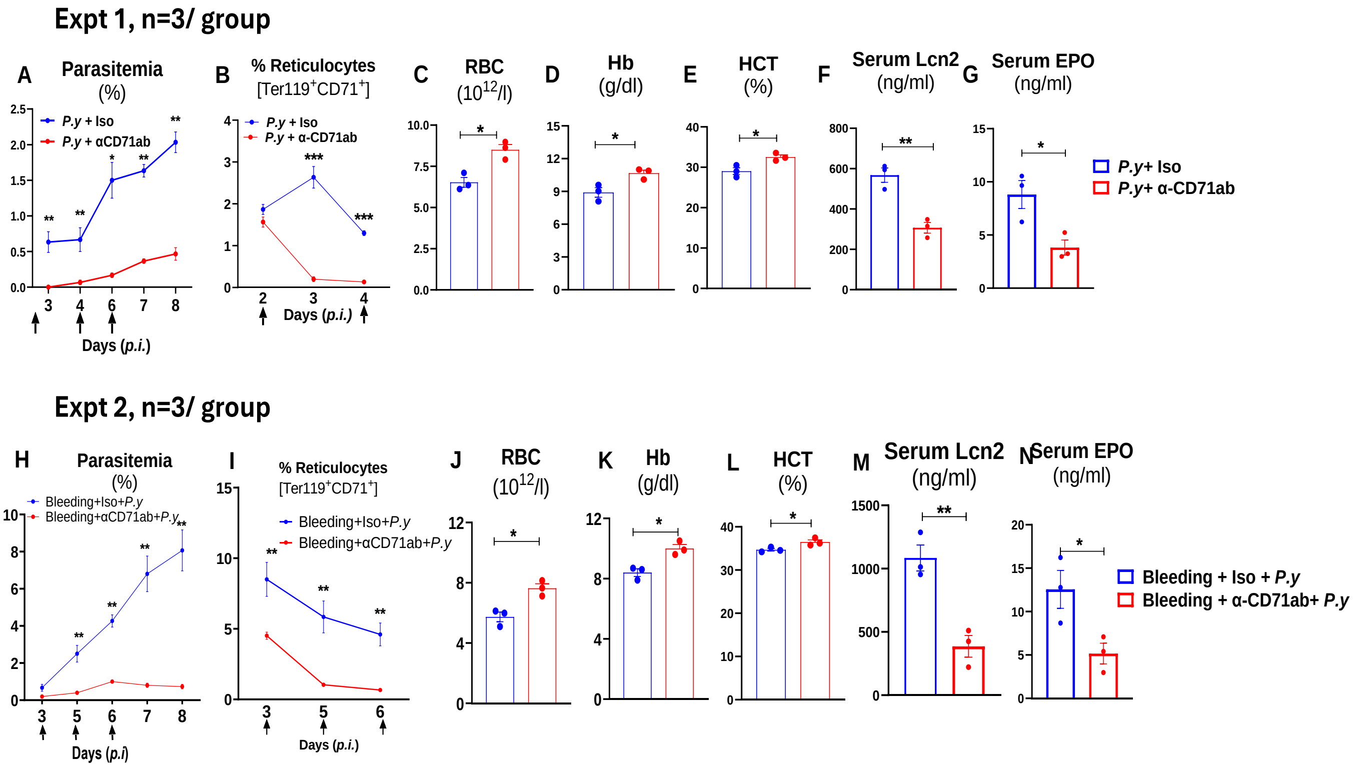

Supplementary Figure 14: Depletion of CD71$^+$ reticulocytes protected mice from *P. yoelii* infection.

In Experiment 1, WT mice (8-weeks-old females) were infected with *P. yoelii* and then divided into two groups (n=3/group); one group received anti-CD71 monoclonal antibody (α-CD71 mAb, TfR, 200 µg/mouse) on days 2, 4, and 6 *p.i.* and the other group received isotype IgG. The mice were euthanized on day 8 *p.i.* **(A)** % Parasitemia. **(B)** % Reticulocytes. CBC analysis day 8 *p.i.* Results for: **(C)** RBC, **(D)** Hb, **(E)** HCT. Serum cytokines, **(F)** Lcn2 and **(G)** EPO were measured by ELISA. Data represented as mean ± SEM. *p<0.05, **p<0.01.

In Experiment 2, bleeding-induced anemic mice (WT, 8-week-old females) were infected with *P. yoelii* 24 h after a blood draw of approximately 200 µl. The mice were divided into two groups (n=3/ group); one group received either α-CD71 mAb (TfR1) or isotype Ab on day 3,5 and 6 *p.i.* The mice were euthanized on day 8 *p.i.* **(H)** % Parasitemia. **(I)** % Reticulocytes. CBC analysis day 8 *p.i.* Results for: **(J)** RBC, **(K)** Hb, **(L)** HCT. Serum cytokines, **(M)** Lcn2 and **(N)** EPO were measured by ELISA. Data represented as mean ± SEM. *p<0.05, **p<0.01.

Supplementary Figure 15

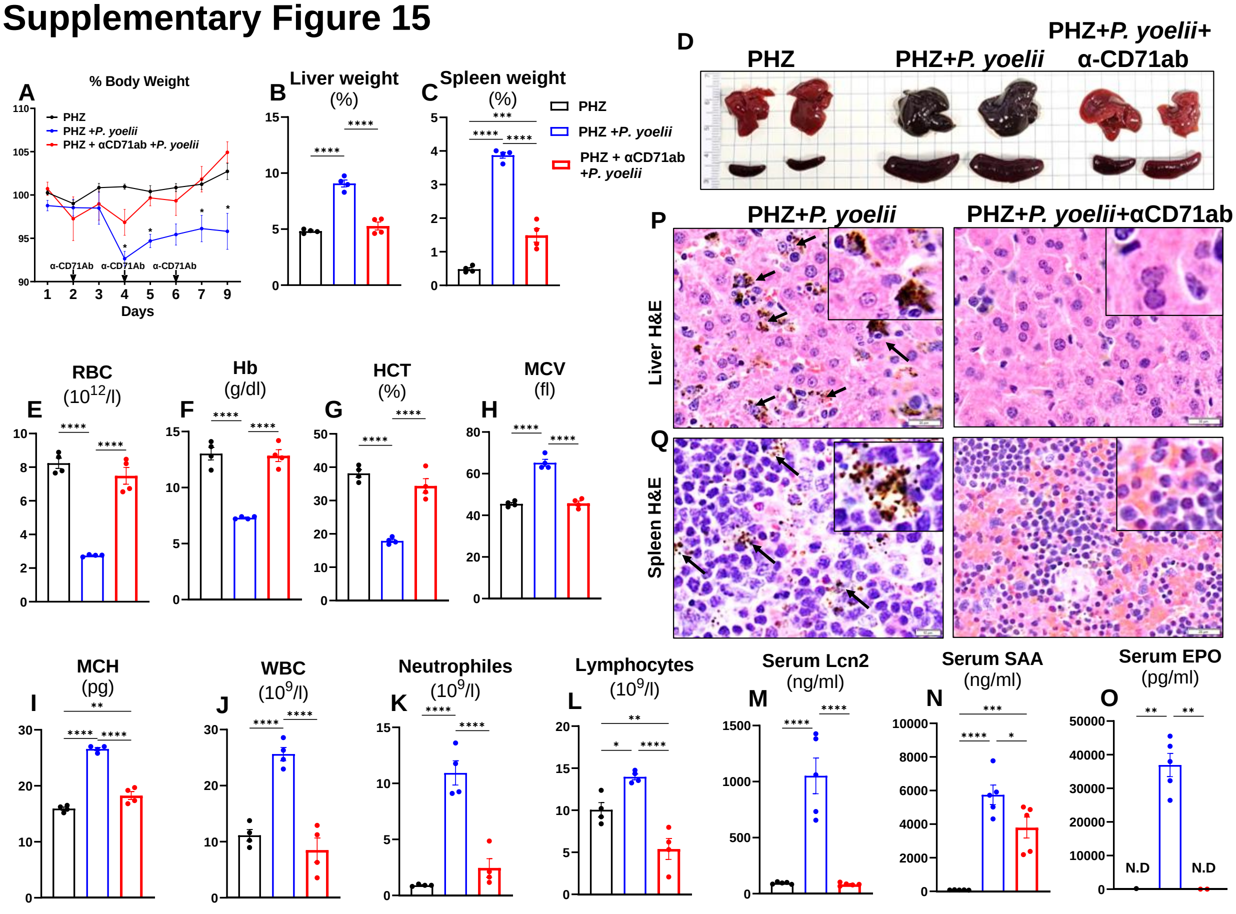

Supplementary Figure 15: Depletion of CD71$^+$ reticulocytes protected PHZ-induced anemic mice from *P. yoelii* infection.

Six-week-old WT male mice were administered PHZ (60 mg/kg) to induce anemia. At 24 h post-PHZ treatment, mice were divided into three groups (n=4/ group): (1) PBS-treated, (2) α-CD71 mAb (TfR1, 200 μg/mouse)-treated, and (3) isotype-treated. The latter two groups were infected with *P. yoelii* 24 h after the first antibody administration. The mice were euthanized on day 9 *p.i.* **(A)** % body weight, **(B)** % Liver weight, **(C)** % Spleen weight. **(D)** Gross organ (liver and spleen) picture. Blood samples were analyzed for CBC. Results for: **(E)** RBC, **(F)** Hb, **(G)** HCT, **(H)** MCV, **(I)** MCH, **(J)** WBC, **(K)** Neutrophils **(L)** Lymphocytes. Serum samples were analyzed for cytokines, **(M)** Lcn2, **(N)** SAA, and **(O)** EPO. Liver and spleen sections were processed for H&E staining to assess histopathological changes. **(P)** Liver and **(Q)** spleen histology depicted at 40X magnification: bars = 20 μm. Black arrows indicate PRBCs and hemozoin pigment sequestration. Inset (60X magnification): Hemozoin pigment (liver and spleen). Data represented as mean ± SEM *p<0.05, **p<0.01 and ***p<0.001, ****p<0.0001.
